# Supplementary material for: Single-atom nanozymes catalytically surpassing naturally occurring enzymes as sustained stitching for brain trauma
Source: Nat Commun. 2022 Aug 12;13:4744. doi: 10.1038/s41467-022-32411-z (PMC9374753; doi:10.1038/s41467-022-32411-z)
Supplement: Supplementary file 1 — Supplementary Information [file 41467_2022_32411_MOESM1_ESM.pdf]

Supplementary Information

Single-Atom Nanozymes Catalytically Surpassing Naturally-occurring Enzymes as Sustained Stitching for Brain Trauma

*Shaofang Zhang[1,†], Yonghui Li[1,†], Si Sun[1,†], Ling Liu[2], Xiaoyu Mu[2], Shuhu Liu[3], Menglu Jiao[1], Xinzhu Chen[2], Ke Chen[2], Huizhen Ma[1], Tuo Li[4], Xiaoyu Liu[1], Hao Wang[2], Jianning Zhang,[4] Jiang Yang[5], Xiao-Dong Zhang [1,2] **

[1] Department of Physics and Tianjin Key Laboratory of Low Dimensional Materials Physics and Preparing Technology, School of Sciences, Tianjin University, Tianjin 300350, China.

[2] Tianjin Key Laboratory of Brain Science and Neural Engineering, Academy of Medical Engineering and Translational Medicine, Tianjin University, Tianjin 300072, China

[3] Beijing Synchrotron Radiation Facility (BSRF), Institute of High Energy Physics (IHEP), Chinese Academy of Sciences (CAS), Beijing, 100049, China

[4] Department of Neurosurgery and Key Laboratory of Post-trauma Neuro-repair and Regeneration in Central Nervous System, Tianjin Medical University General Hospital, Tianjin 300052, China

[5] State Key Laboratory of Oncology in South China, Collaborative Innovation Center for Cancer Medicine, Sun Yat-sen University Cancer Center, Guangzhou 510060, China

*Correspondence to Xiaodong Zhang, email: xiaodongzhang@tju.edu.cn.

†: These authors contributed equally.

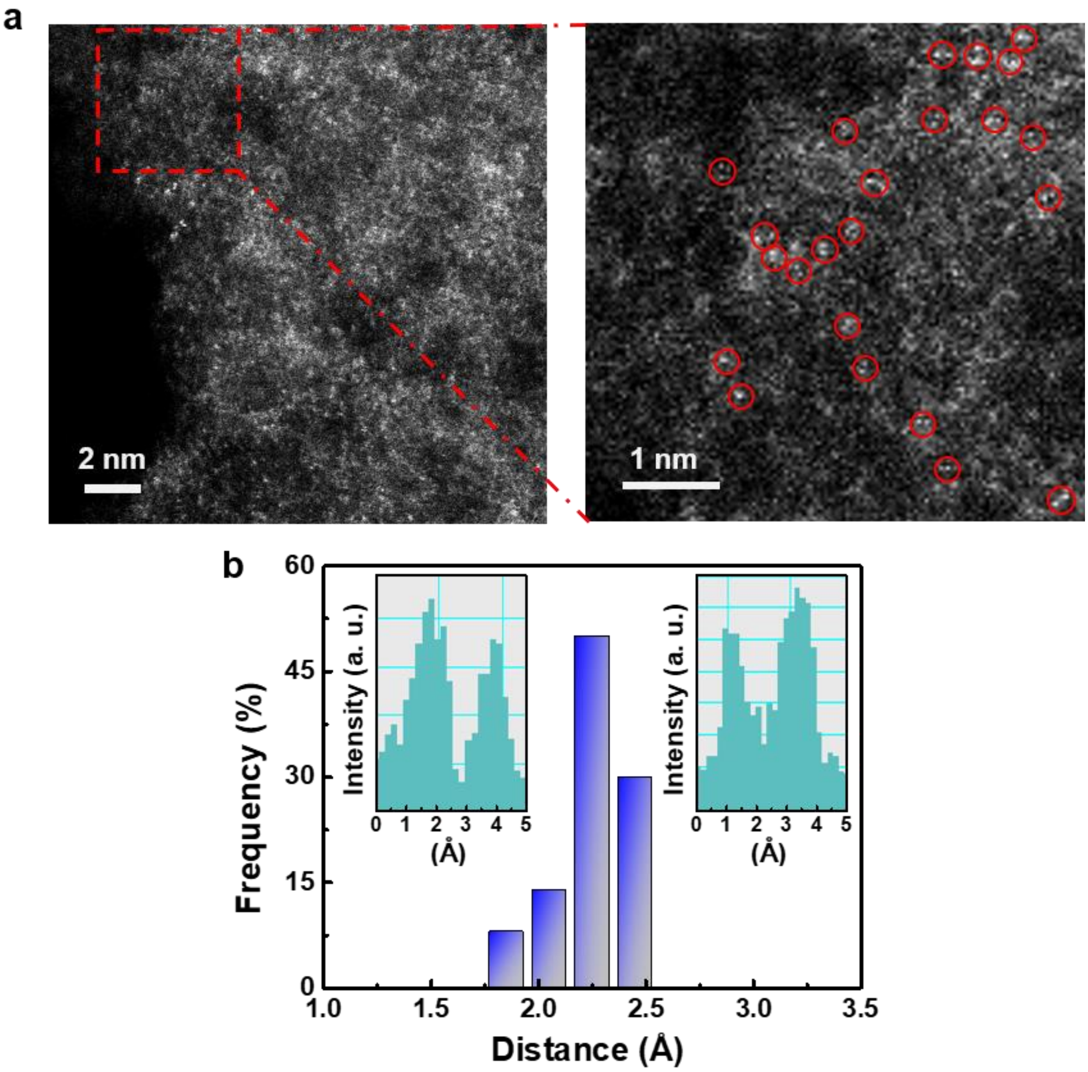

Supplementary Fig. 1. Structural characterization of Fe-Cu-N_6. **a**, The magnified AC-HAADF-STEM image of Fe-Cu-N_6 (n=3 images from three independent samples). Diatomic pairs highlighted by red circles. **b**, The corresponding intensity distributions and the statistical Fe-Cu distance of the observed diatomic pairs.

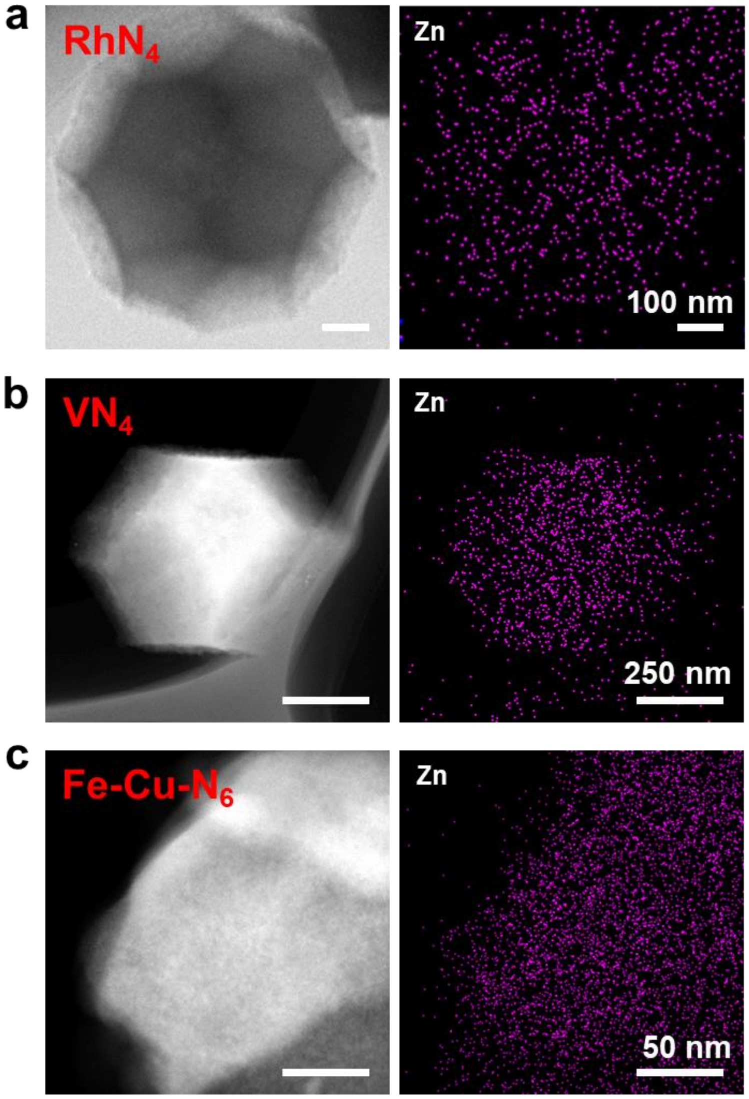

Supplementary Fig. 2. Energy-dispersive X-ray spectroscopy (EDS) mapping images of (**a**) RhN_4, (**b**) VN_4, and (**c**) Fe-Cu-N_6, respectively (n=3 images from three independent samples).

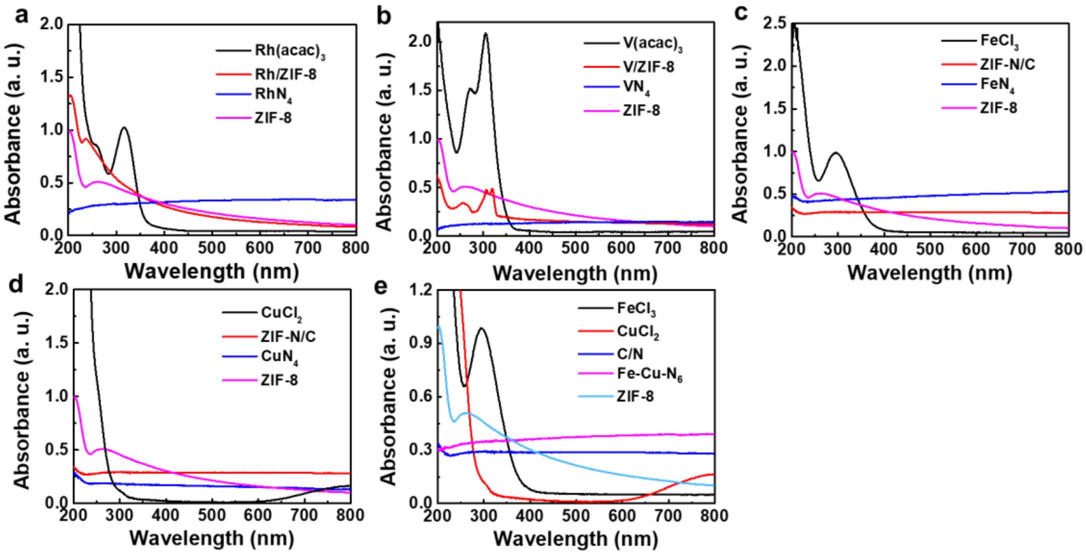

Supplementary Fig. 3. UV-vis spectra of metal precursors and intermediates of (**a**) RhN_4, (**b**) VN_4, and (**c**) FeN_4, (**d**) CuN_4, and (**e**) Fe-Cu-N_6. ‘a. u.’ represents arbitrary units.

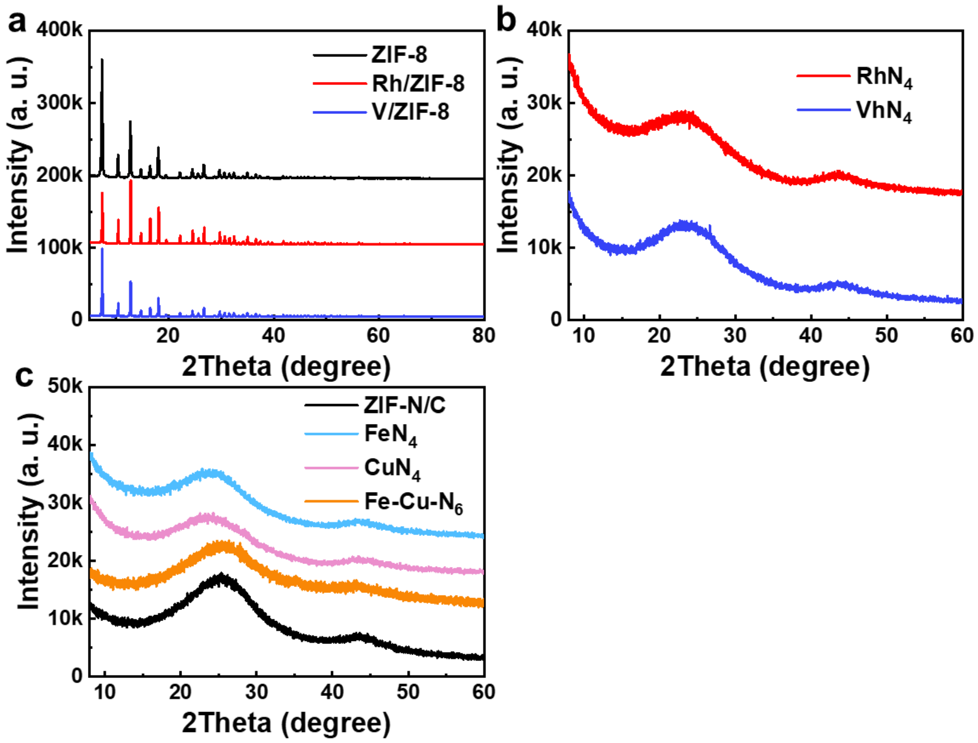

Supplementary Fig. 4. Powder X-ray diffraction (XRD) patterns of (**a**) ZIF-8, Rh/ZIF-8, and V/ZIF-8, (**b-c**) MN_x, and (**c**) ZIF-N/C, where ZIF-N/C and MN_x were prepared by annealing at 900 ºC in the N_2 atmosphere. The results suggest that the addition of metal salts preserved the ZIF-8 crystalline structure, and the two characteristic peaks at 20-30° and 40-50° represent the presence of amorphous carbon. 'a. u.' represents arbitrary units.

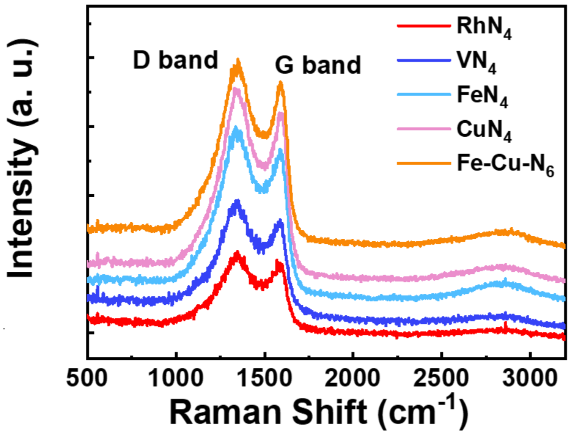

Supplementary Fig. 5. The Raman spectra of MN_x with two dominant peaks located at 1345 cm^{-1} (D band) and 1586 cm^{-1} (G band) suggest the graphitic C structure in MN_x. 'a. u.' represents arbitrary units.

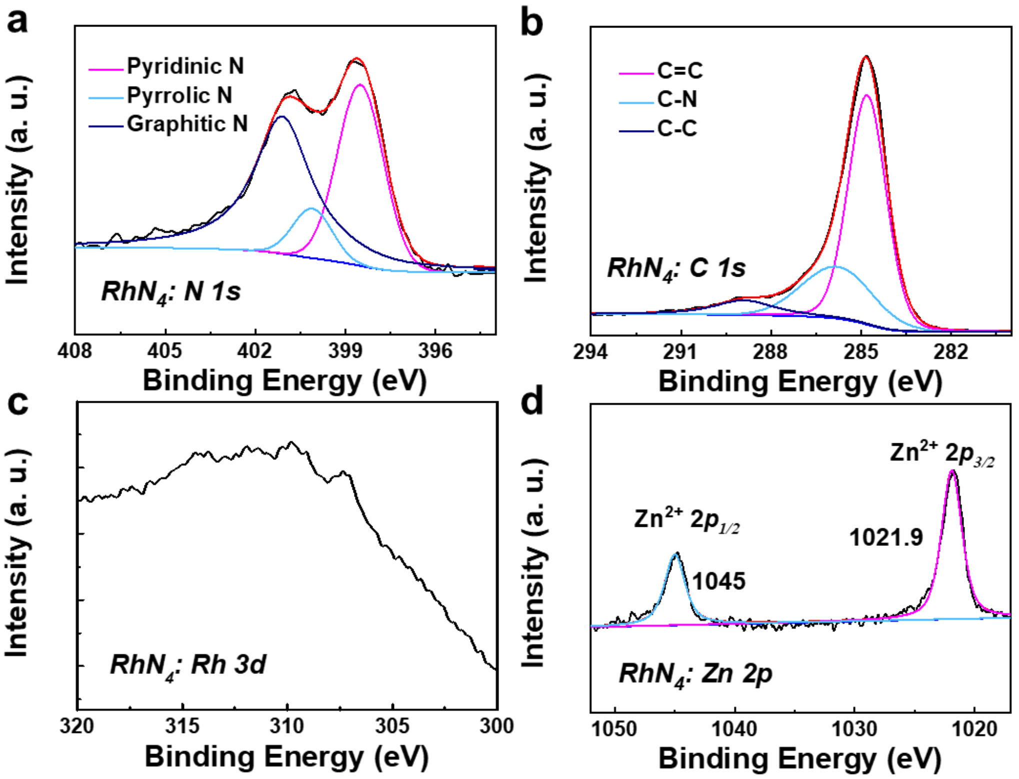

Supplementary Fig. 6. X-ray photoelectron spectra (XPS) of RhN_4 in (**a**) N *1s*, (**b**) C *1s*, (**c**) Rh 3*d*, and (**d**) Zn 2*p* regions. 'a. u.' represents arbitrary units.

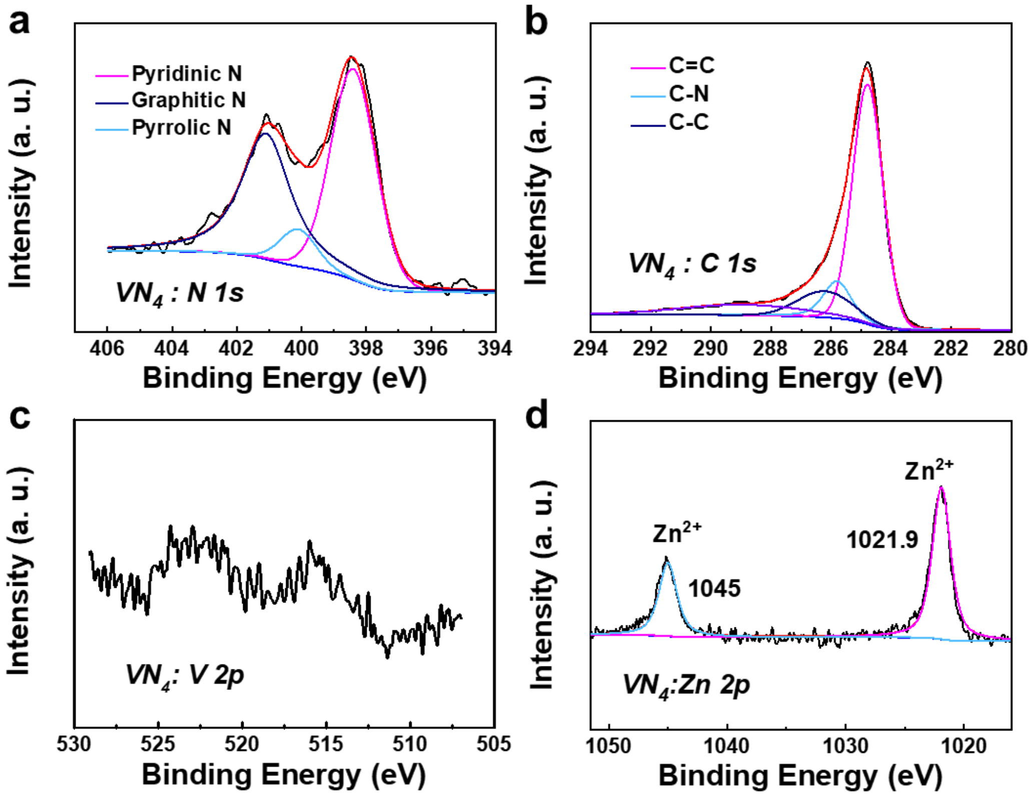

Supplementary Fig. 7. XPS spectra of VN_4 in (**a**) N *1s*, (**b**) C *1s*, (**c**) V *2p*, and (**d**) Zn *2p* regions. 'a. u.' represents arbitrary units.

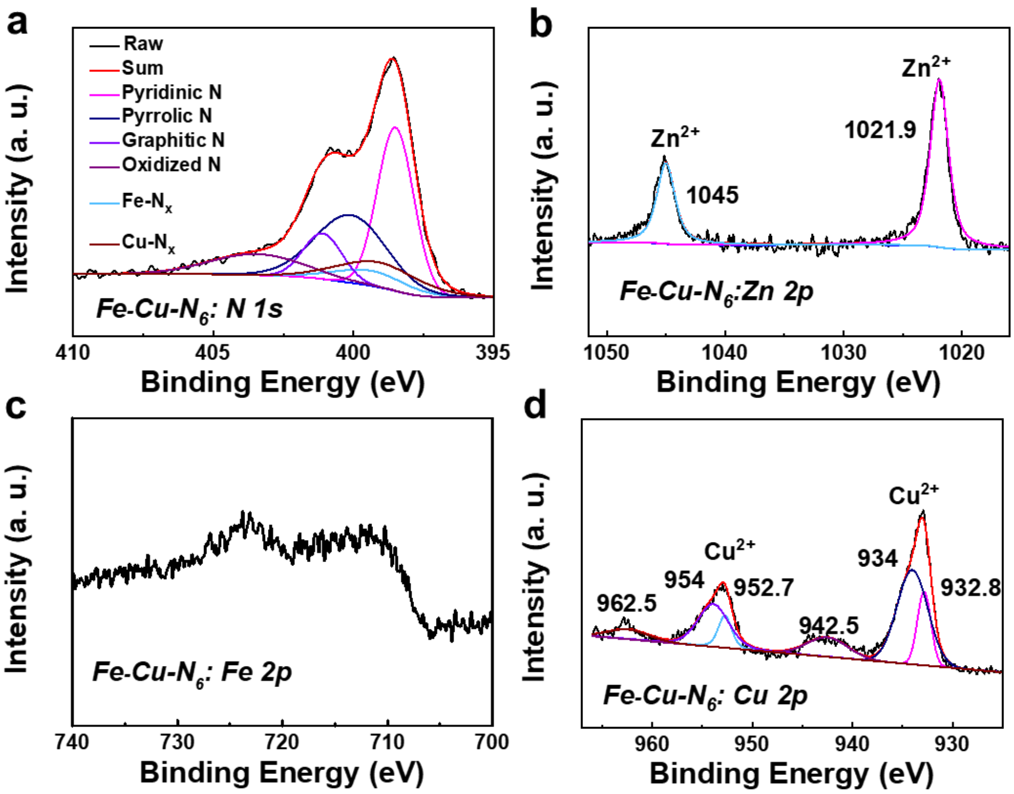

Supplementary Fig. 8. XPS spectra of Fe-Cu-N_6 in (**a**) N *1s*, (**b**) Zn *2p*, (**c**) Fe 2*p*, and (**d**) Cu 2*p* regions. 'a. u.' represents arbitrary units.

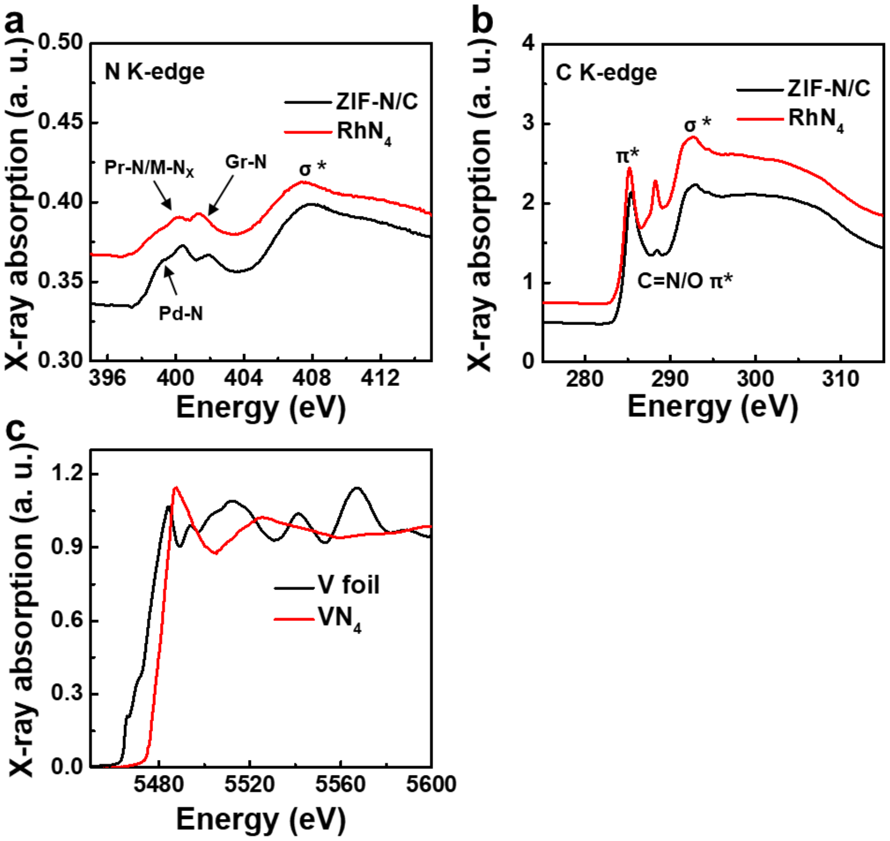

Supplementary Fig. 9. XAFS analysis of RhN_4 and VN_4. (**a**) N K-edge and (**b**) C K-edge XANES spectra of RhN_4 and the N/C reference. **c**, V K-edge XANES spectra of VN_4 and the V foil reference. The Pr-N, Gr-N, and Pd-N represent pyrrolic N, graphitic N, and pyridinic N. 'a. u.' represents arbitrary units.

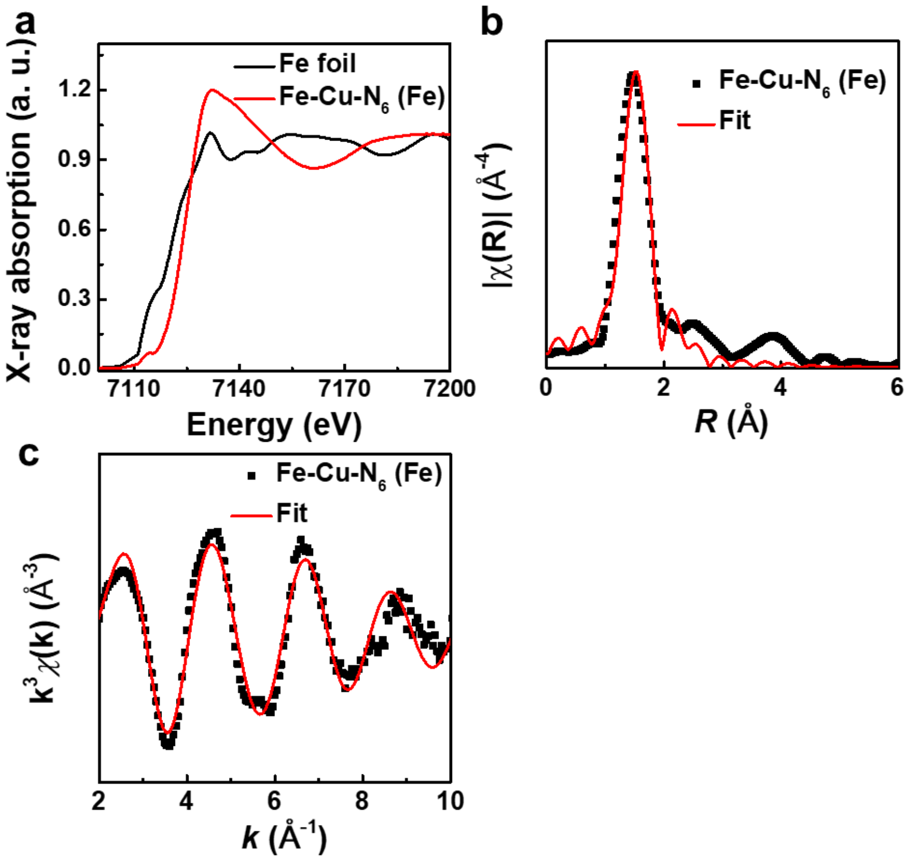

Supplementary Fig. 10. XAFS analysis of the Fe element for Fe-Cu-N_6. **a**, Fe K-edge XANES spectra of Fe-Cu-N_6 and the Fe foil reference. The EXAFS (**b**) R and (**c**) k spaces with fitting curves for the Fe K-edge of Fe-Cu-N_6. 'a. u.' represents arbitrary units.

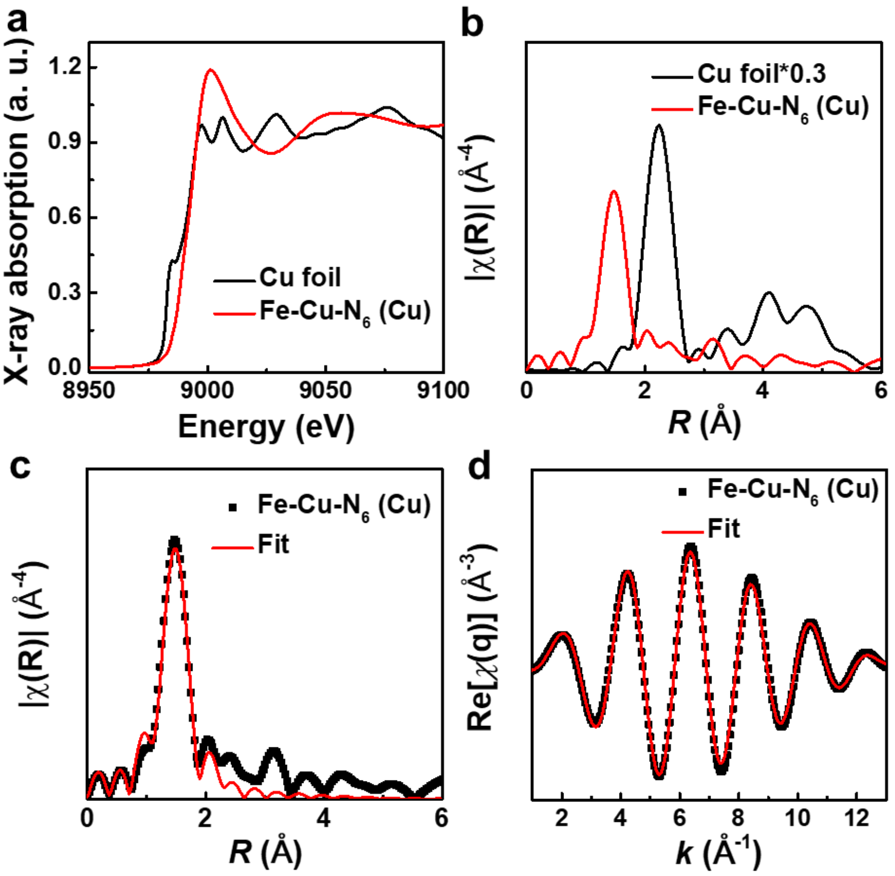

Supplementary Fig. 11. XAFS analysis of the Cu element for Fe-Cu-N_6. **a**, The Cu K-edge XANES spectra of Fe-Cu-N_6 and the Cu foil reference. **b**, The Fourier-transformed magnitude of the Cu K-edge EXAFS signal of Fe-Cu-N_6. **c-d**, The EXAFS (**c**) R and (**d**) q spaces with fitting curves of the Cu K-edge for Fe-Cu-N_6. 'a. u.' represents arbitrary units.

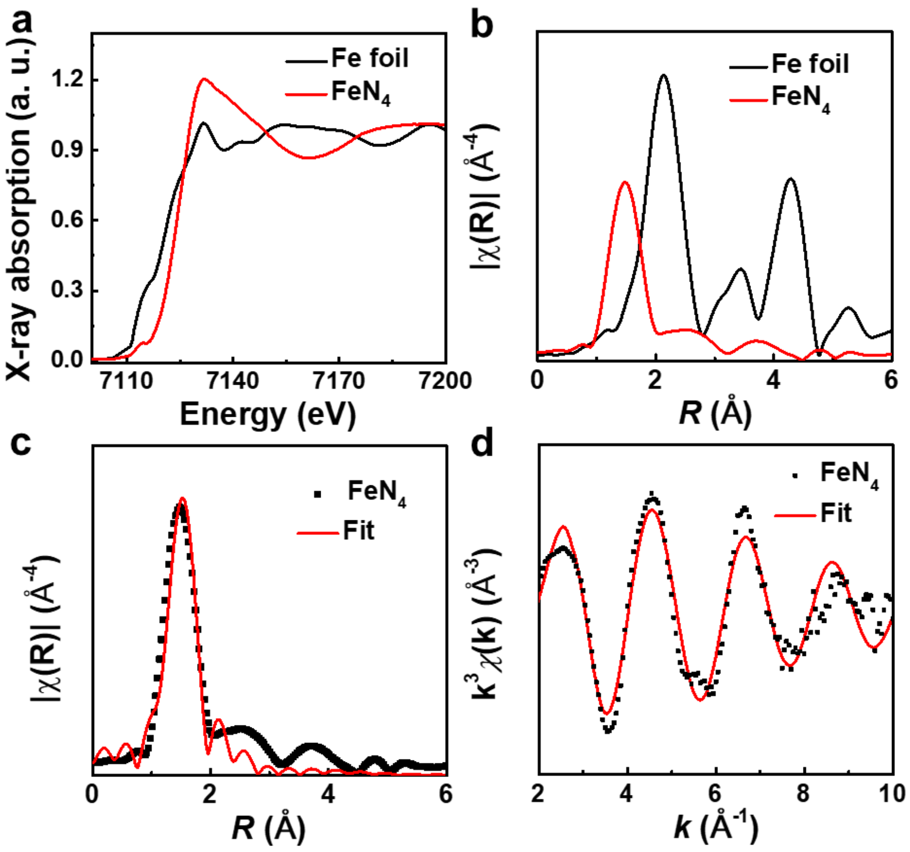

Supplementary Fig. 12. XAFS analysis of FeN_4. **a**, Fe K-edge XANES spectra of FeN_4 and the Fe foil reference. **b**, The Fourier-transformed magnitude of the Fe K-edge EXAFS signal for FeN_4. **c-d**, The EXAFS R (**c**) and k (**d**) spaces with fitting curves of the Fe K-edge for FeN_4. 'a. u.' represents arbitrary units.

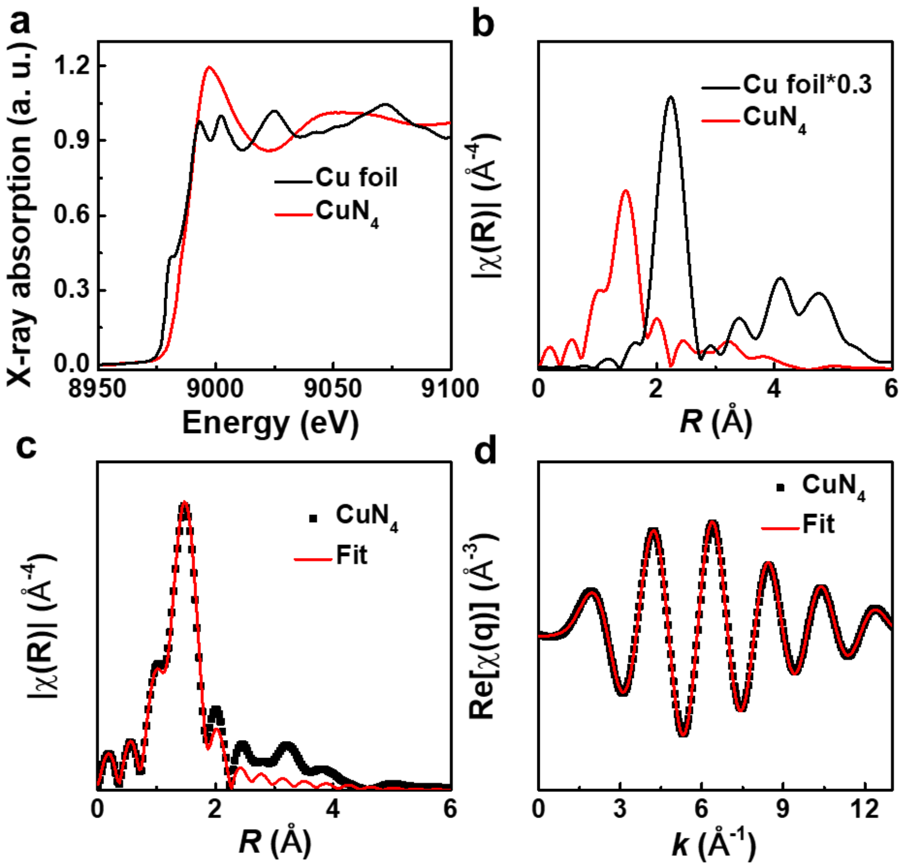

Supplementary Fig. 13. XAFS analysis of CuN_4. **a**, The Cu K-edge XANES spectra of CuN_4 and the Cu foil reference. **b**, The Fourier-transformed magnitude of the Cu K-edge EXAFS signal of CuN_4. **c-d**, The EXAFS (**c**) R and (**d**) q spaces with fitting curves of the Cu K-edge for CuN_4. ‘a. u.’ represents arbitrary units.

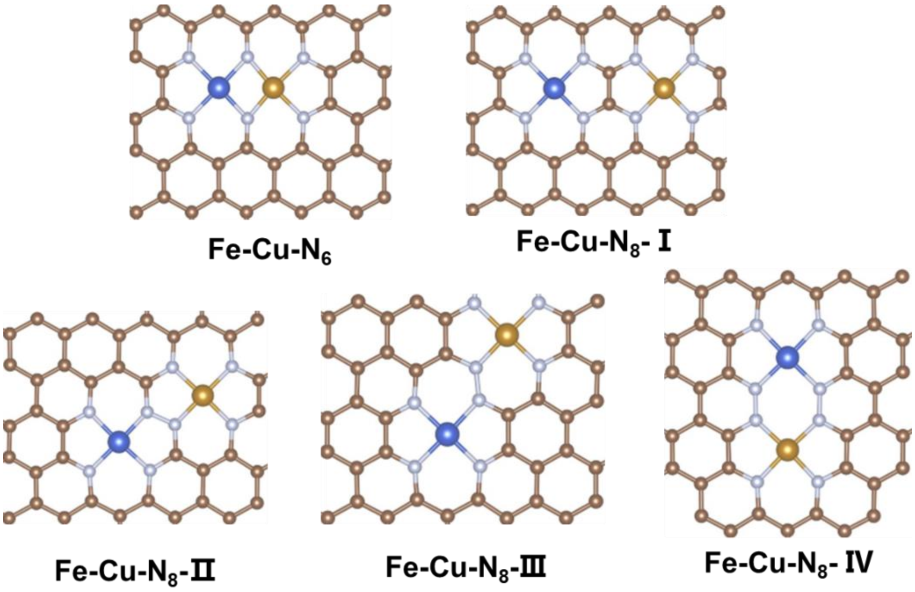

Supplementary Fig. 14. The optimized structures of five different Fe-Cu-N_x structures.

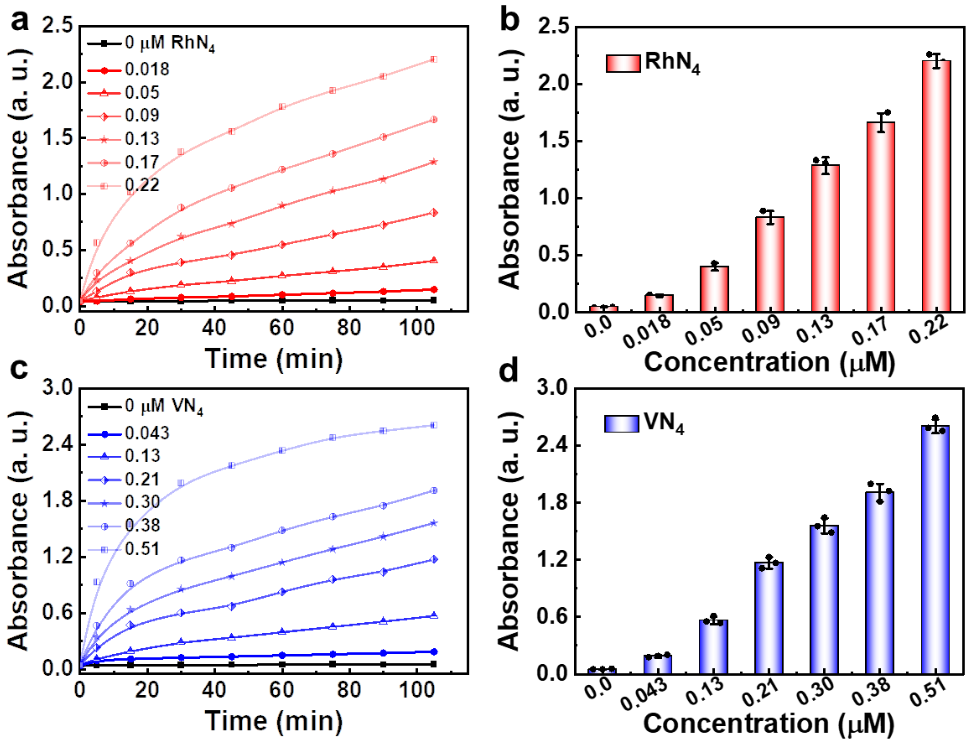

Supplementary Fig. 15. The POD-like activity of MN_x. Concentration-dependent POD-like activities of (**a-b**) RhN_4 and (**c-d**) VN_4 (n=3 independent experiments, data are presented as mean ± SD). 'a. u.' represents arbitrary units.

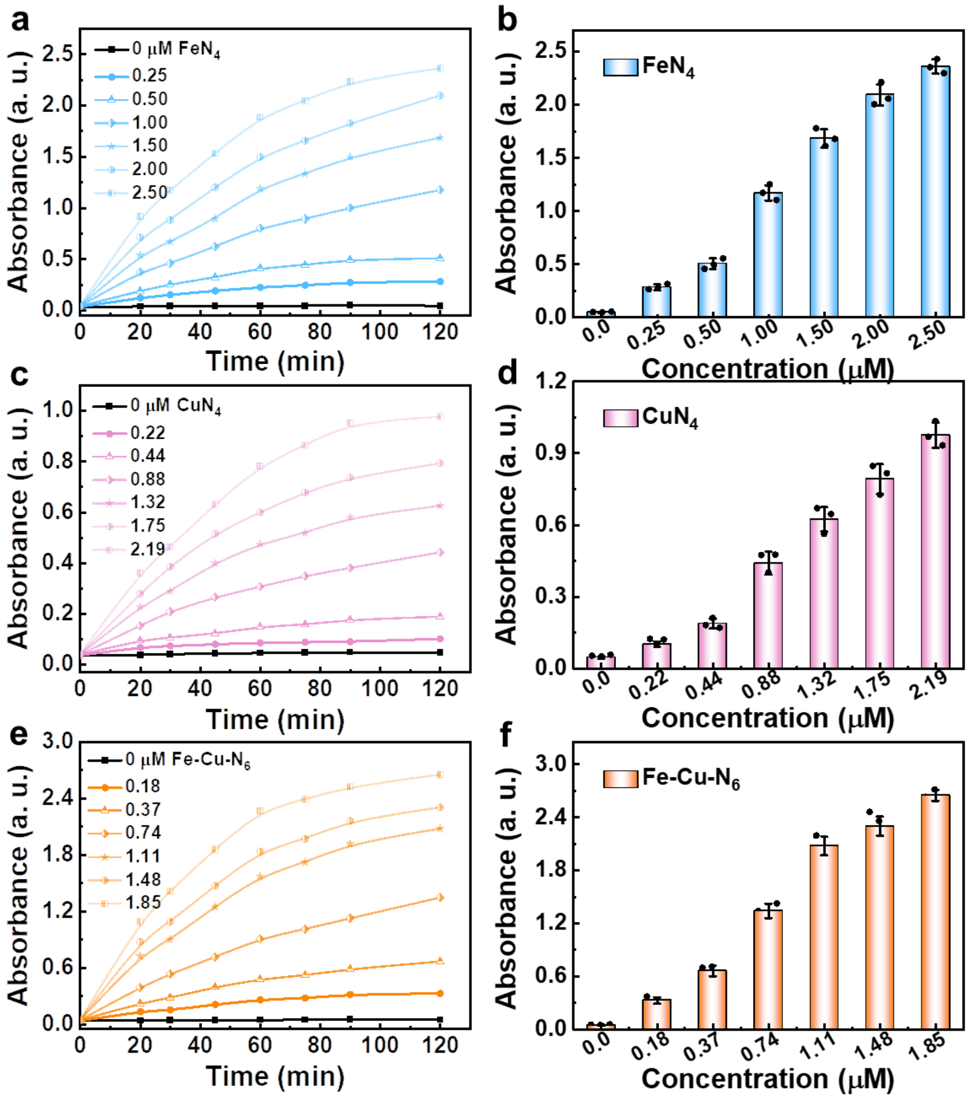

Supplementary Fig. 16. The POD-like activity of MN_x. Concentration-dependent POD-like activities of (**a-b**) FeN_4, (**c-d**) CuN_4 and (**e-f**) Fe-Cu-N_6 (n=3 independent experiments, data are presented as mean ± SD). 'a. u.' represents arbitrary units.

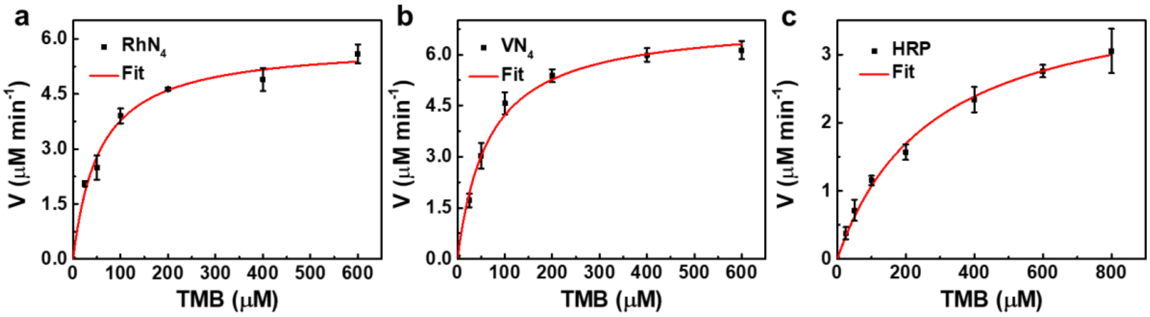

Supplementary Fig. 17. Steady-state kinetic analysis of POD-like activities. The Michaelis-Menten plots at various concentrations of the TMB substrate (0-800 μM) for (**a**) RhN_4, (**b**) VN_4, and (**c**) HRP (n=3 independent experiments, data are presented as mean ± SD).

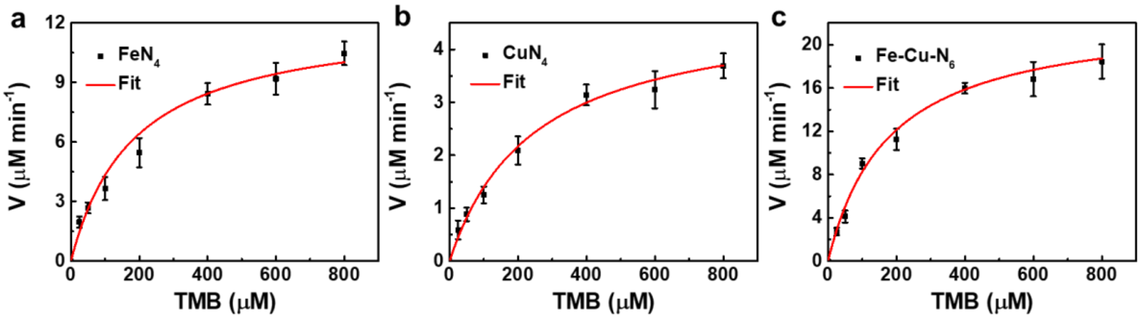

Supplementary Fig. 18. Steady-state kinetic analysis of POD-like activities. The Michaelis-Menten and corresponding Lineweaver-Burk plots at various concentrations of the TMB substrate (0-800 μM) for (**a**) FeN_4, (**b**) CuN_4, and (**c**) Fe-Cu-N_6 (n=3 independent experiments, data are presented as mean ± SD).

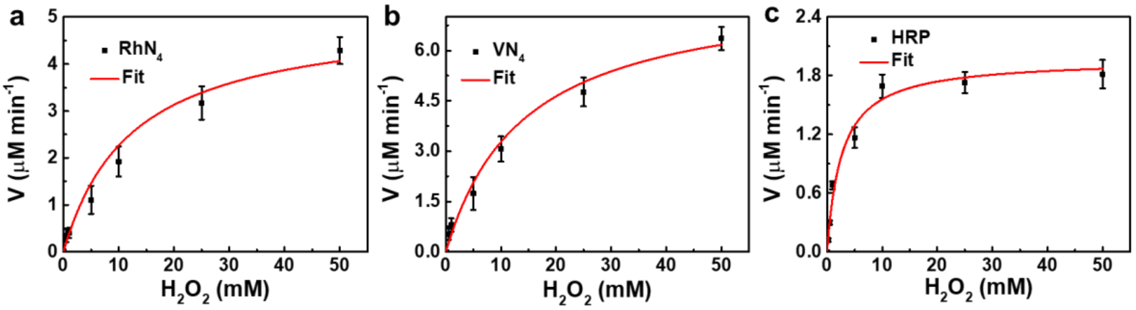

Supplementary Fig. 19. Steady-state kinetic analysis of POD-like activities. The Michaelis-Menten and corresponding Lineweaver-Burk plots at various concentrations of H_2O_2 (0-50 mM) for (**a**) RhN_4, (**b**) VN_4, and (**c**) HRP (n=3 independent experiments, data are presented as mean ± SD).

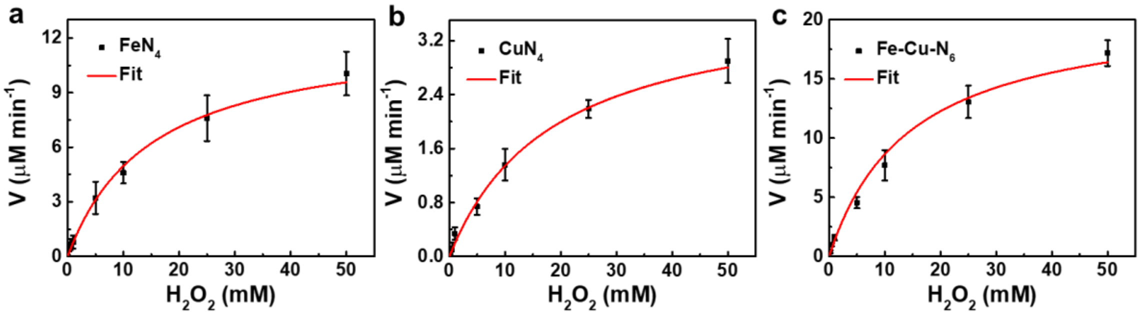

Supplementary Fig. 20. Steady-state kinetic analysis of POD-like activities. The Michaelis-Menten and corresponding Lineweaver-Burk plots at various concentrations of H_2O_2 (0-50 mM) for (**a**) FeN_4, (**b**) CuN_4, and (**c**) Fe-Cu-N_6 (n=3 independent experiments, data are presented as mean ± SD).

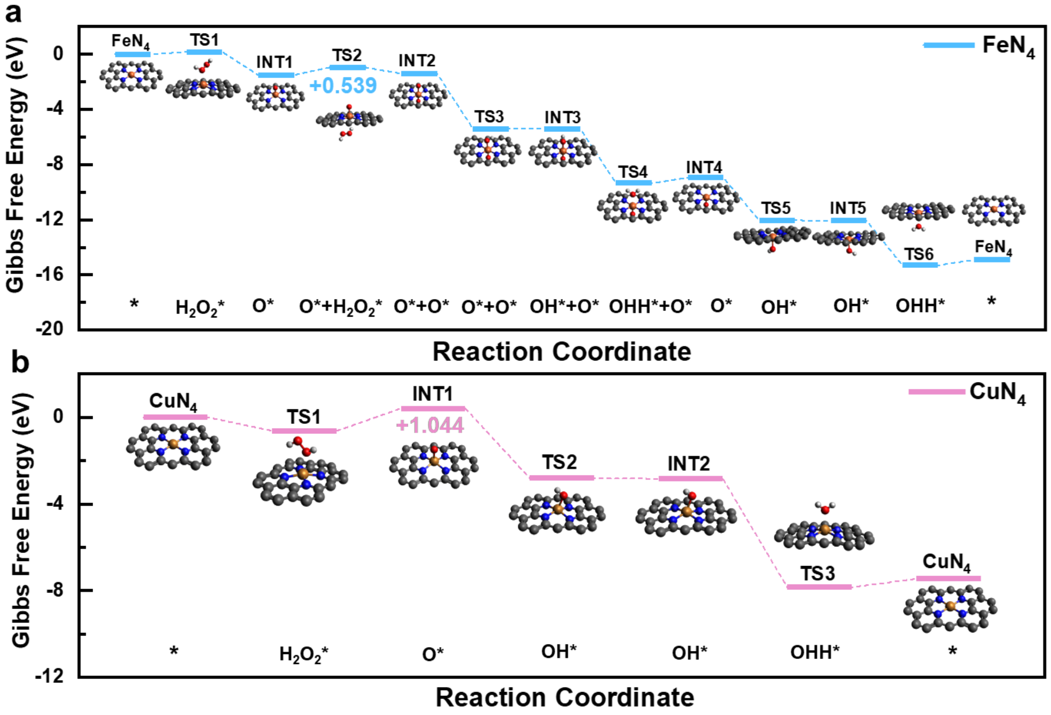

Supplementary Fig. 21. The simulated POD processes for (**a**) FeN_4 and (**b**) CuN_4.

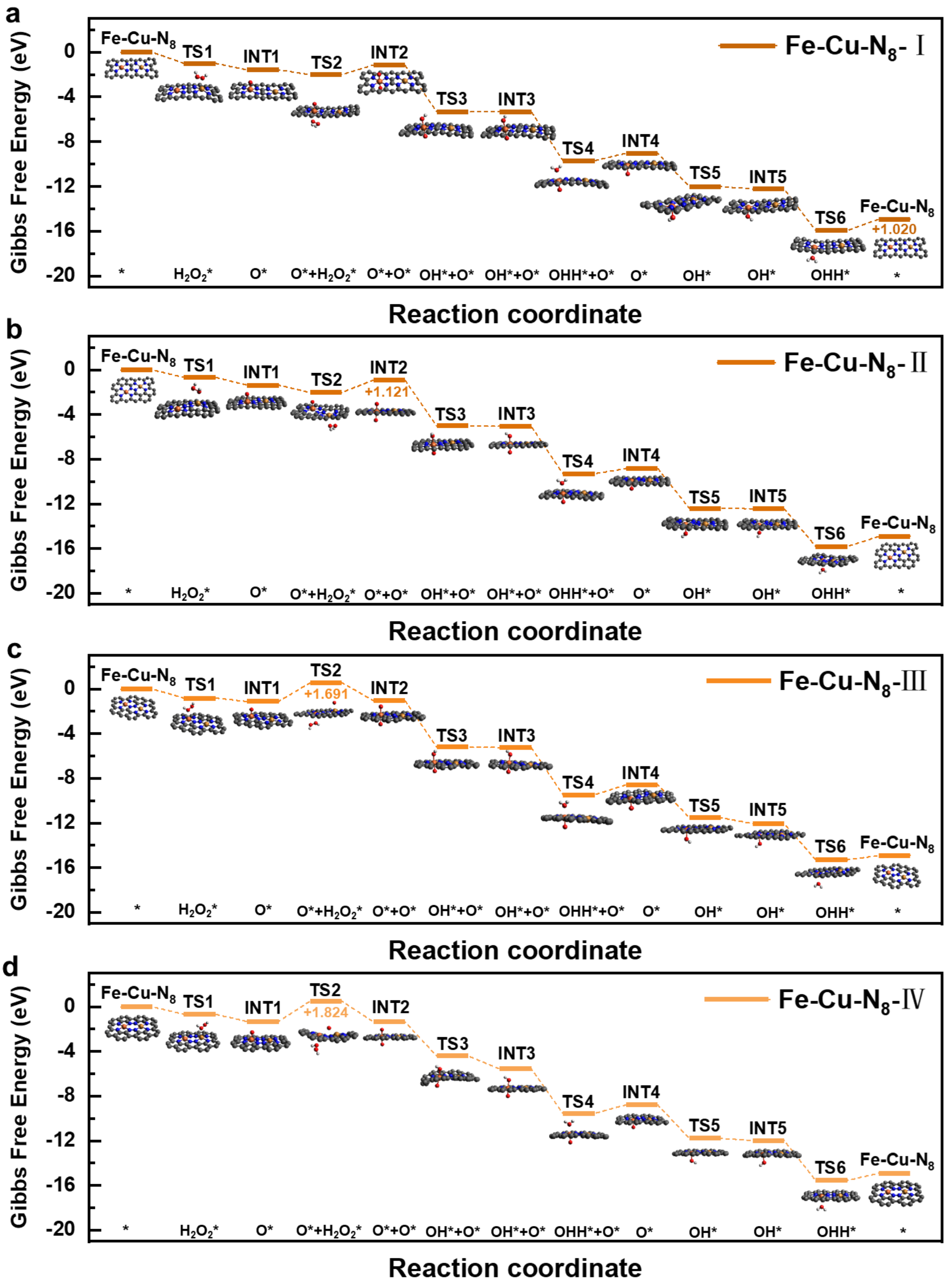

Supplementary Fig. 22. POD processes with (**a**) Fe-Cu-N_8-I, (**b**) Fe-Cu-N_8-II, (**c**) Fe-Cu-N_8-III and (**d**) Fe-Cu-N_8- IV.

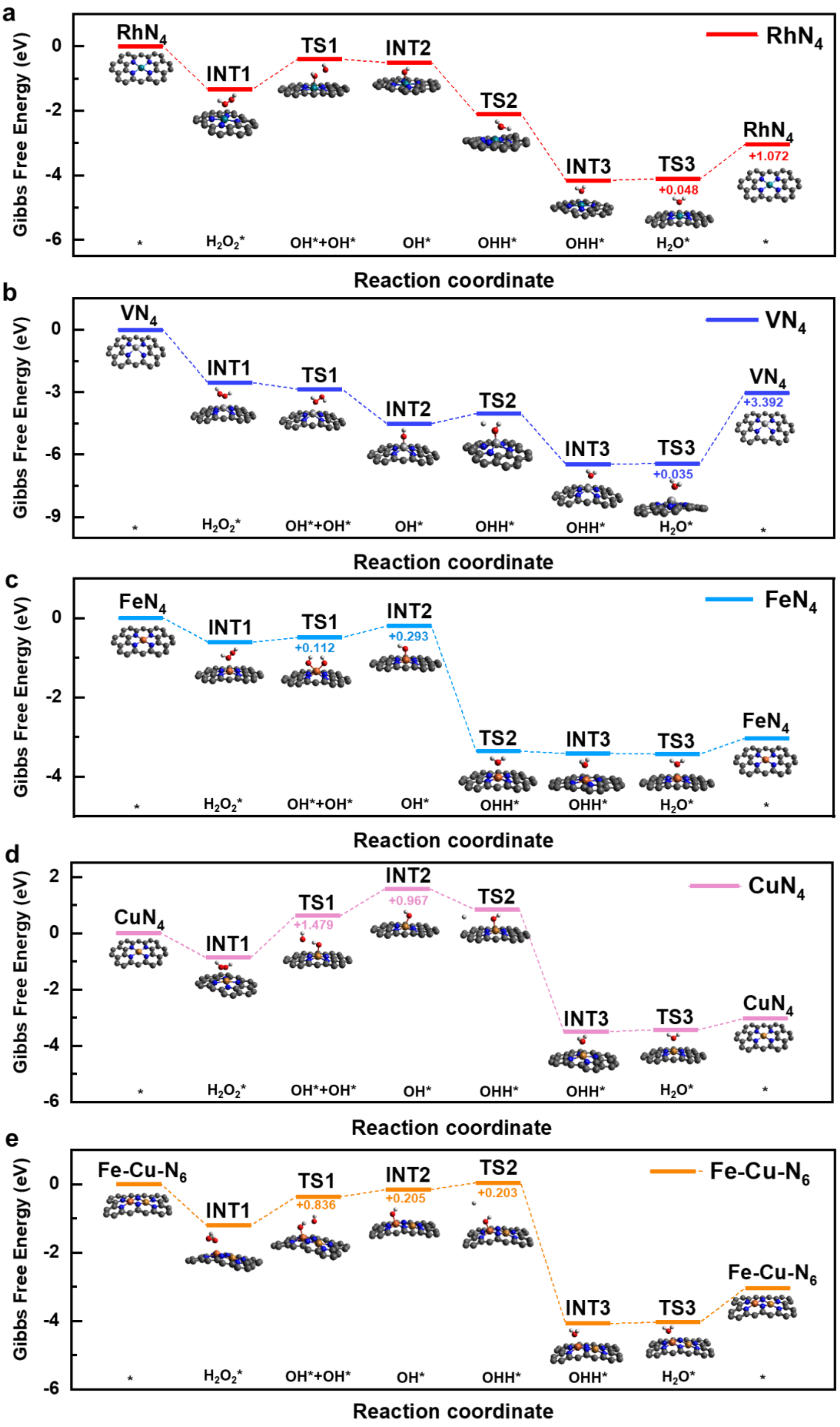

Supplementary Fig. 23. POD processes for generating $^{\bullet}OH$ with (**a**) RhN_4, (**b**) VN_4, (**c**) FeN_4, (**d**) CuN_4 and (**e**) Fe-Cu-N_6.

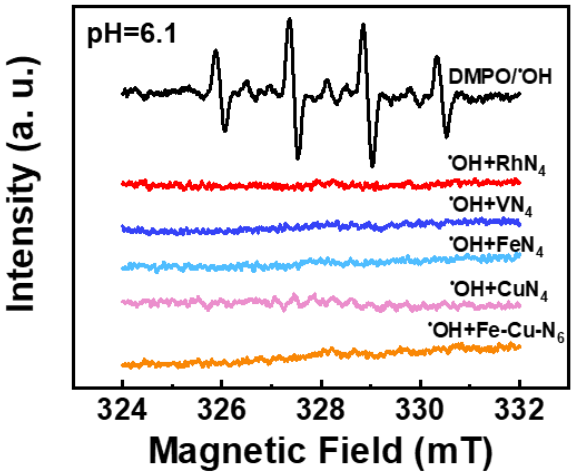

Supplementary Fig. 24. The •OH scavenging ability of MN_x at 100 ng/μL using DMPO as the radical-scavenging nitrogen trap. 'a. u.' represents arbitrary units.

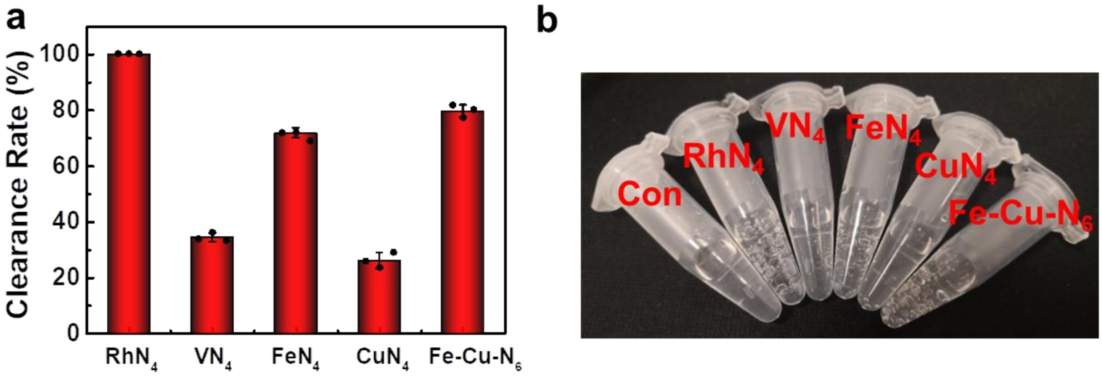

Supplementary Fig. 25. The CAT-like activity of MN_x. **a**, The H_2O_2 clearance rates of MN_x (n=3 independent experiments, data are presented as mean ± SD). **b**, Photographs of generated O_2 bubbles resulting from catalysis by MN_x. 50 mM of H_2O_2 (400 μL) and 1.25 μM of MN_x were incubated for 3 min for reactions.

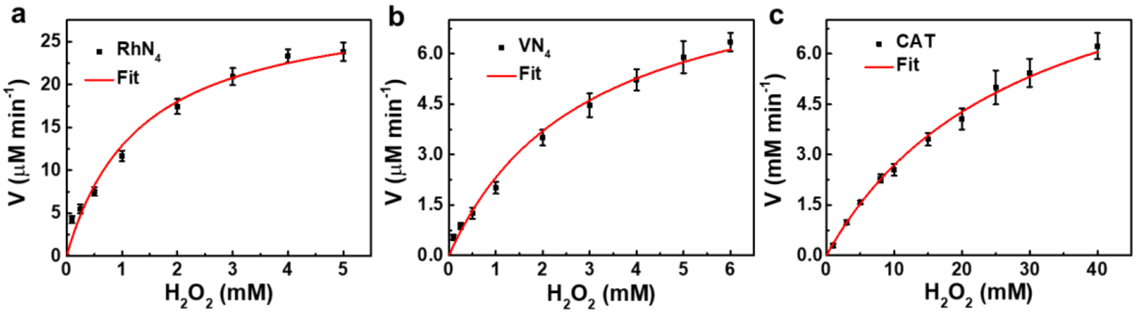

Supplementary Fig. 26. Steady-state kinetic profiles of the CAT-like activities. The Michaelis-Menten and corresponding Lineweaver-Burk plots at various concentrations of H_2O_2 (0-5 mM) for (**a**) RhN_4, (**b**) VN_4, and (**c**) CAT (n=3 independent experiments, data are presented as mean ± SD).

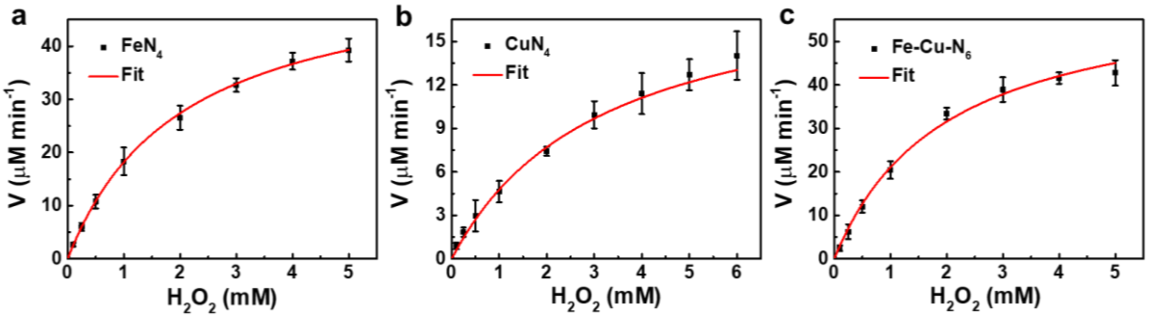

Supplementary Fig. 27. Steady-state kinetic profiles of the CAT-like activities. The Michaelis-Menten and corresponding Lineweaver-Burk plots at various concentrations of H_2O_2 (0-5 mM) for (**a**) FeN_4, (**b**) CuN_4, and (**c**) Fe-Cu-N_6 (n=3 independent experiments, data are presented as mean ± SD).

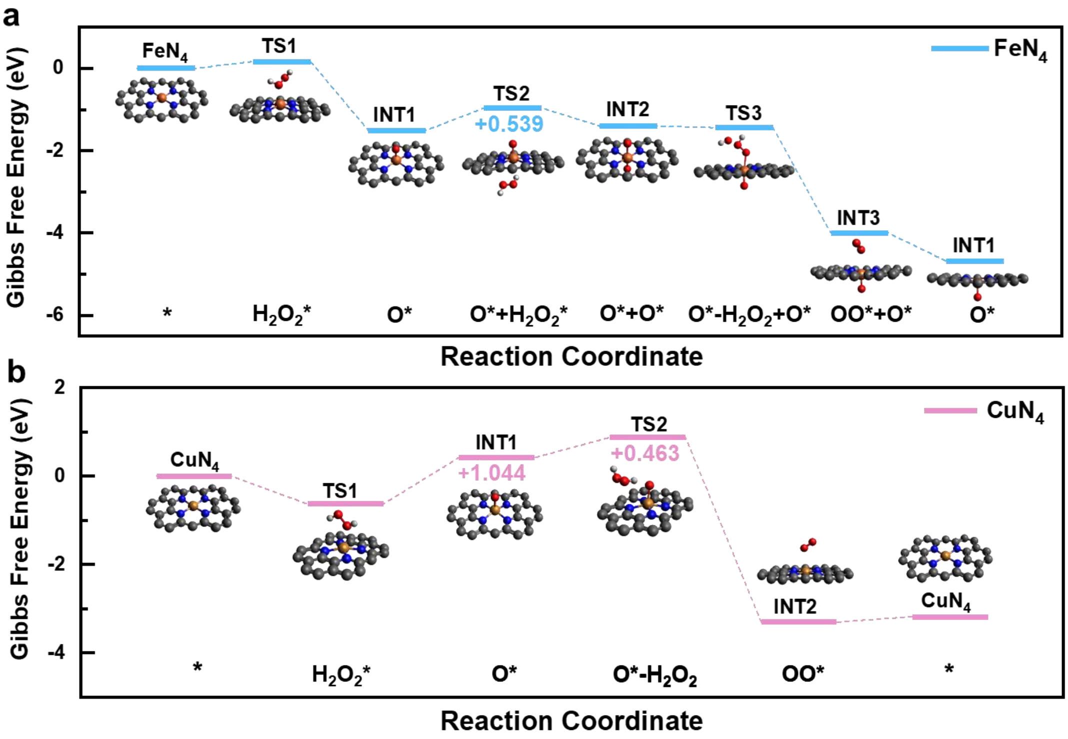

Supplementary Fig. 28. The simulated CAT processes for (**a**) FeN_4 and (**b**) CuN_4.

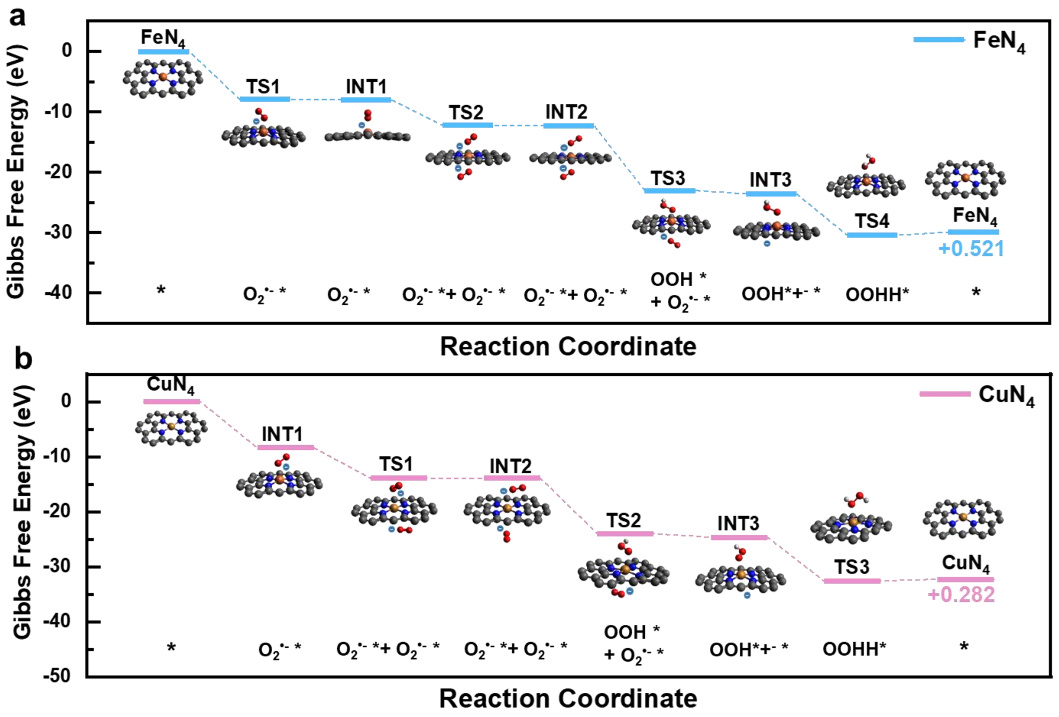

Supplementary Fig. 29. The simulated SOD processes for (**a**) FeN_4 and (**b**) CuN_4.

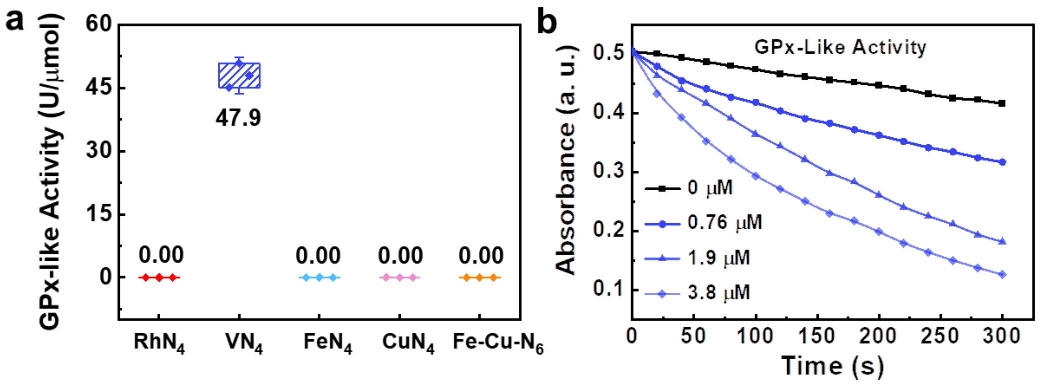

Supplementary Fig. 30. The GPx-like activity of MN_x. **a**, Quantification for the specific GPx-like activity of VN_4 (n=3 independent experiments, boxes represent the median and interquartile range (IQR) and the upper and lower whiskers extending to the values that are within 1.5 × IQR). The specific activity (U/μmol) is determined by dividing the GPx-like activity by the metal-based active sites. **b**, Correlation of the GPx-like activity of VN_4 with concentrations. 'a. u.' represents arbitrary units.

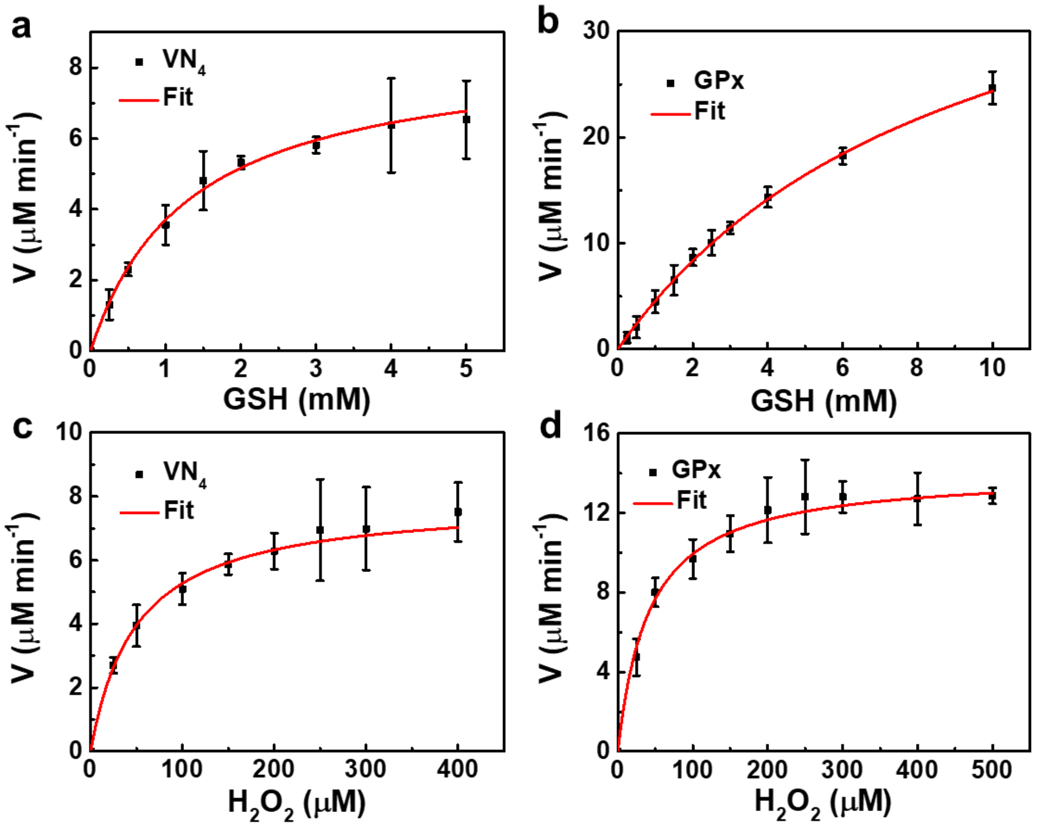

Supplementary Fig. 31. Steady-state kinetic profiles of the GPx-like activity for VN_4 and GPx. The Michaelis-Menten plot at various concentrations of (**a-b**) GSH at 0-5 mM and (**c-d**) H_2O_2 at 0-500 μM (n=3 independent experiments, data are presented as mean ± SD).

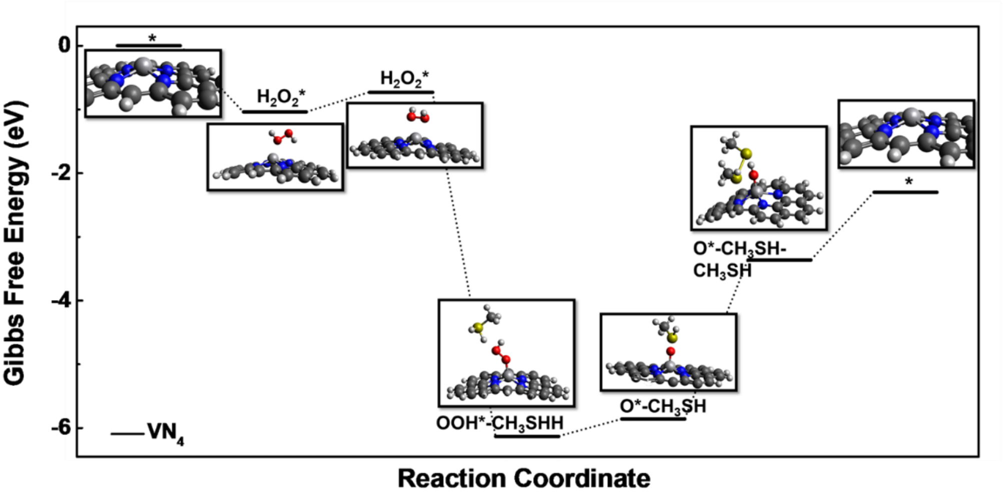

Supplementary Fig. 32. The catalysis process of GPx for VN_4.

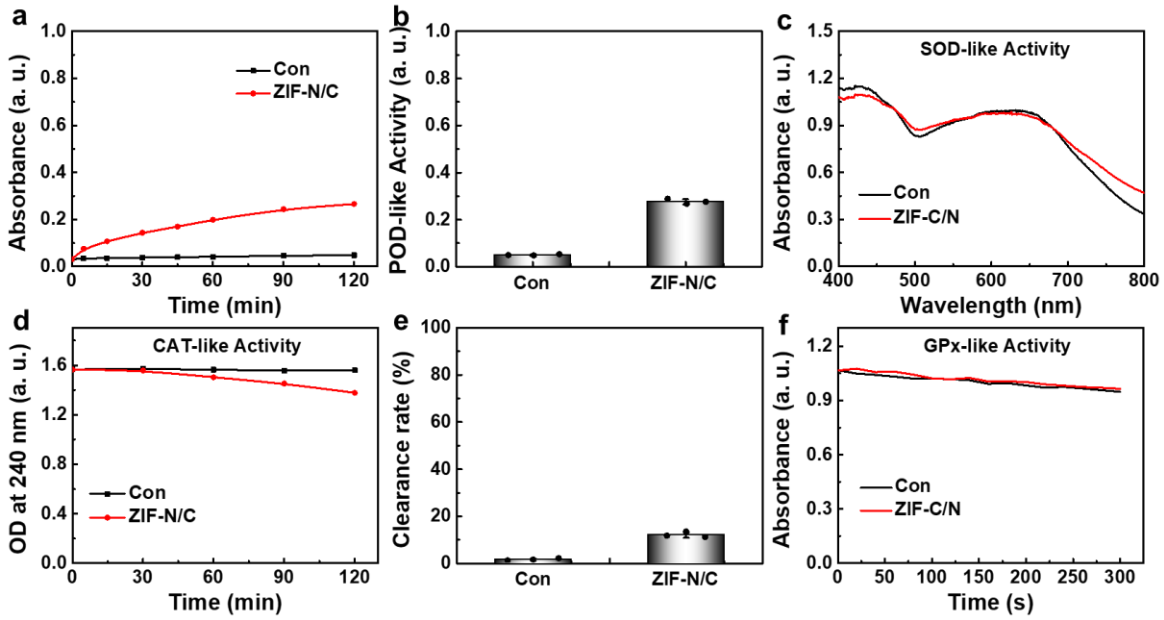

Supplementary Fig. 33. The enzyme-like activities of ZIF-N/C**. a**, Absorbance-time curves of the TMB chromogenic reaction catalyzed by ZIF-N/C (3 ng/μL). **b**, The POD-like activity of ZIF-N/C (n=3 independent experiments, data are presented as mean ± SD). **c**, SOD-like activity with and without ZIF-N/C (30 ng/μL). **d**, Reaction-time curves of the decomposition of H_2O_2 catalyzed by ZIF-N/C (30 ng/μL). **e**, The corresponding H_2O_2 clearance rate of ZIF-N/C (30 ng/μL) (n=3 independent experiments, data are presented as mean ± SD). **f**, The GPx-like activity with and without ZIF-N/C (30 ng/μL). 'a. u.' represents arbitrary units. Some error bars are too small to be visible.

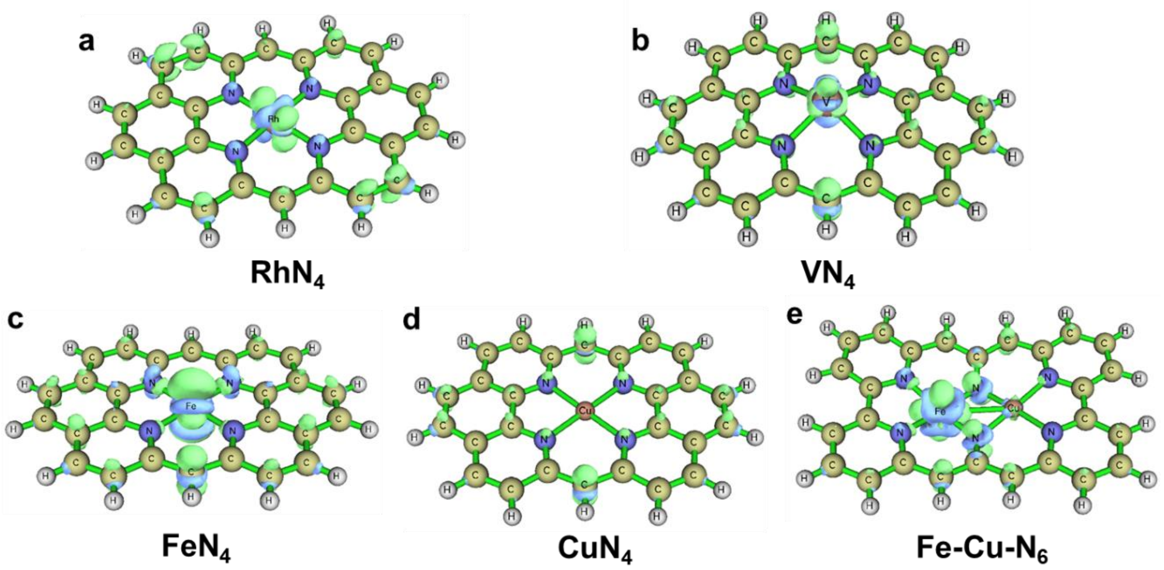

Supplementary Fig. 34. Donor Fukui Function $f^{-}(\vec{\mathbf{r}})$ of (**a**) RhN_4, (**b**) VN_4, (**c**) FeN_4, (**d**) CuN_4, and (**e**) Fe-Cu-N_6. The green and blue areas denote positive and negative of $f^{-}(\vec{\mathbf{r}})$.

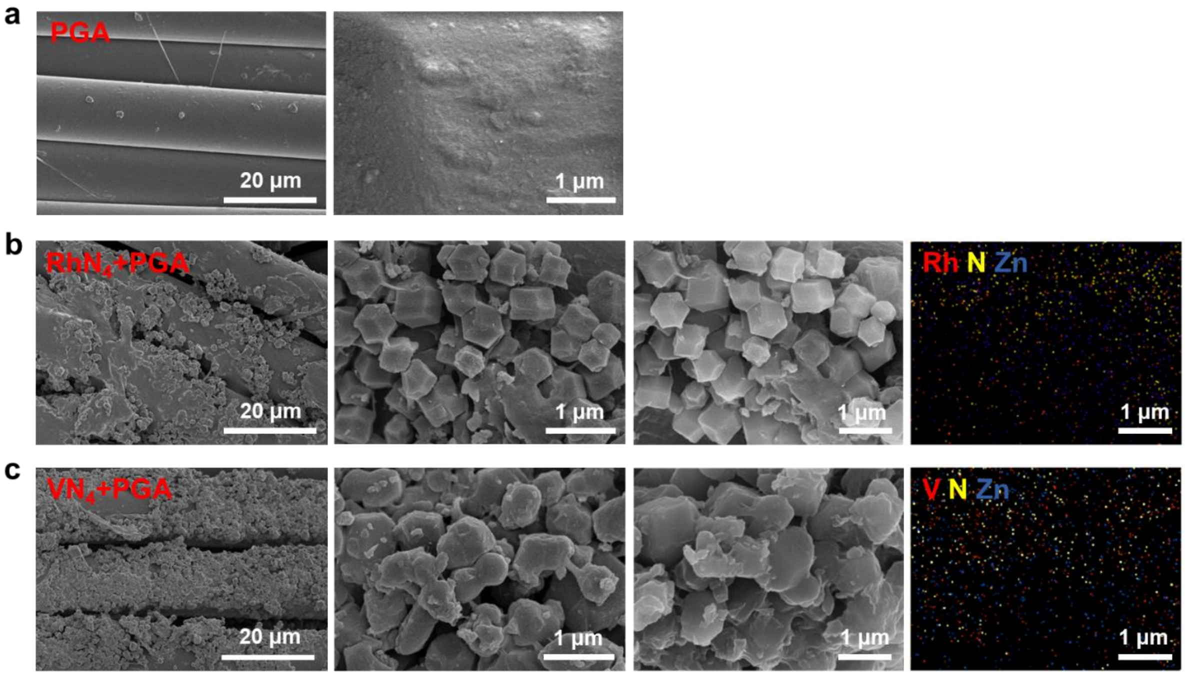

Supplementary Fig. 35. SEM and corresponding EDS mapping images of (**a**) pristine PGA sutures, (**b**) RhN_4, and (**c**) VN_4 sutures. The presence of elemental Rh and V was confirmed in corresponding sutures (n=3 images from three independent samples).

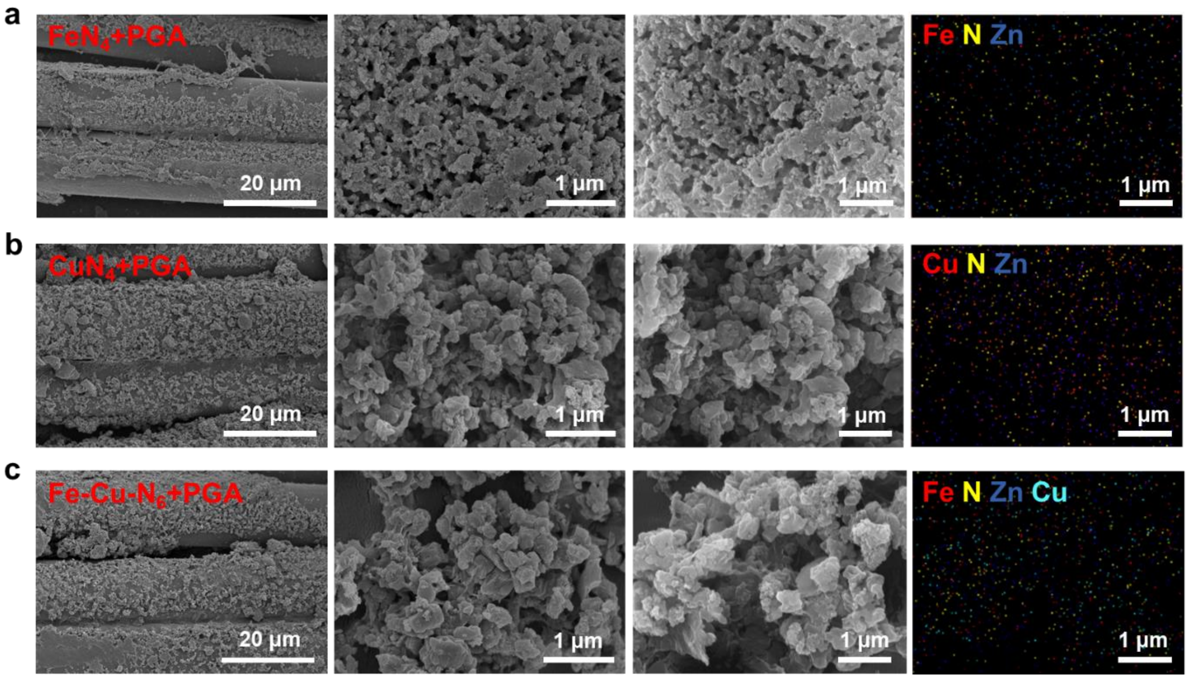

Supplementary Fig. 36. SEM and corresponding EDS mapping images of (**a**) FeN_4, (**b**) CuN_4, and (**c**) Fe-Cu-N_6 sutures. Single or dual elements can be identified in the respective suture structures (n=3 images from three independent samples).

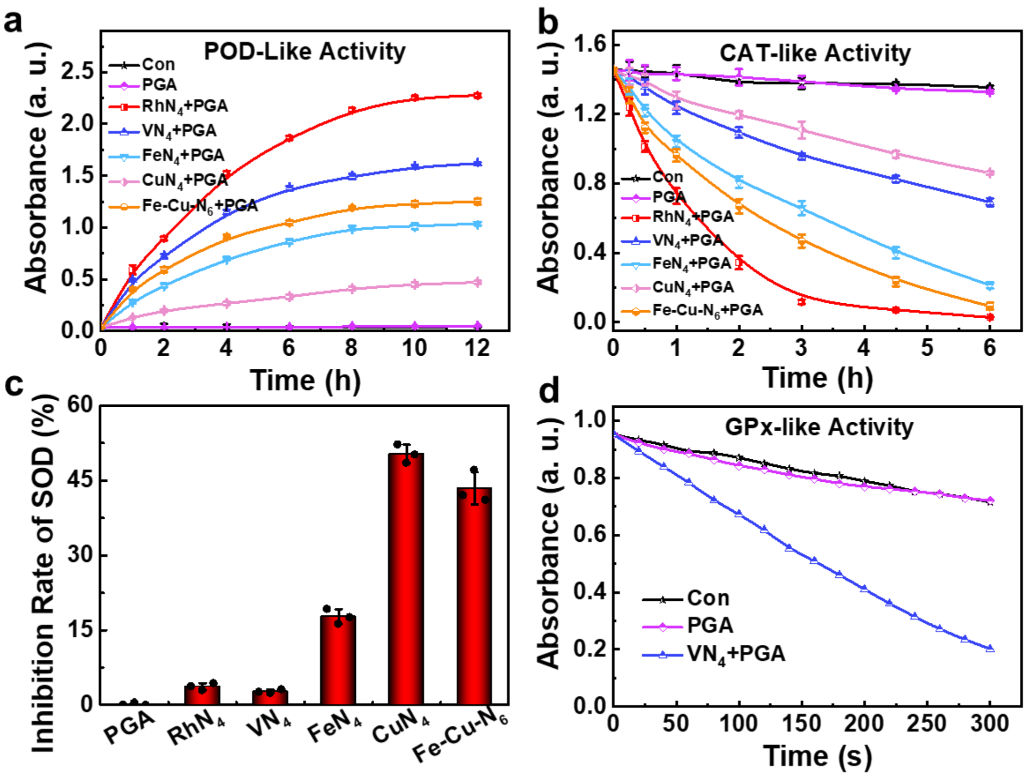

Supplementary Fig. 37. Evaluation of (**a**) POD-, (**b**) CAT-, (**c**) SOD-, and (**d**) GPx-like activities of the native PGA suture and MN_x sutures (n=3 independent experiments, data are presented as mean ± SD). 'a. u.' represents arbitrary units. Some error bars are too small to be visible.

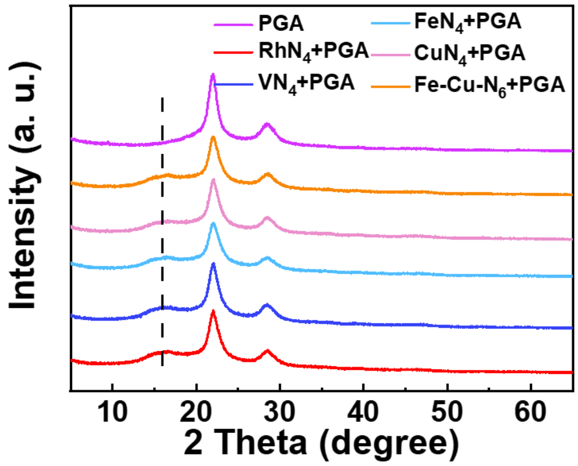

Supplementary Fig. 38. XRD patterns of the native PGA suture and MN_x sutures. The black dashed line indicates characteristic peaks of graphitic C in the intrinsic background signal of the pure PGA. ‘a. u.’ represents arbitrary units.

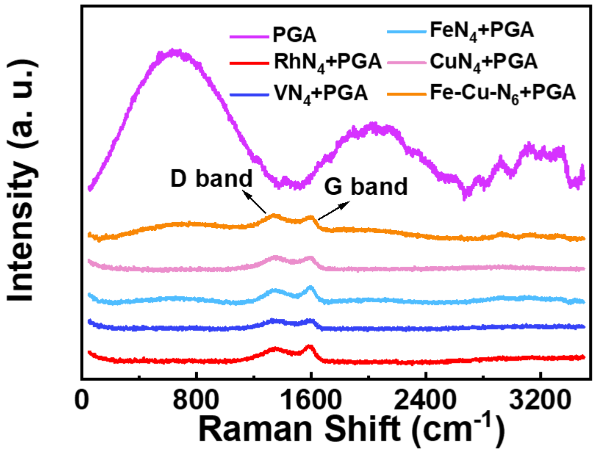

Supplementary Fig. 39. Raman spectra of the native PGA suture and MN_x sutures. ‘a. u.’ represents arbitrary units.

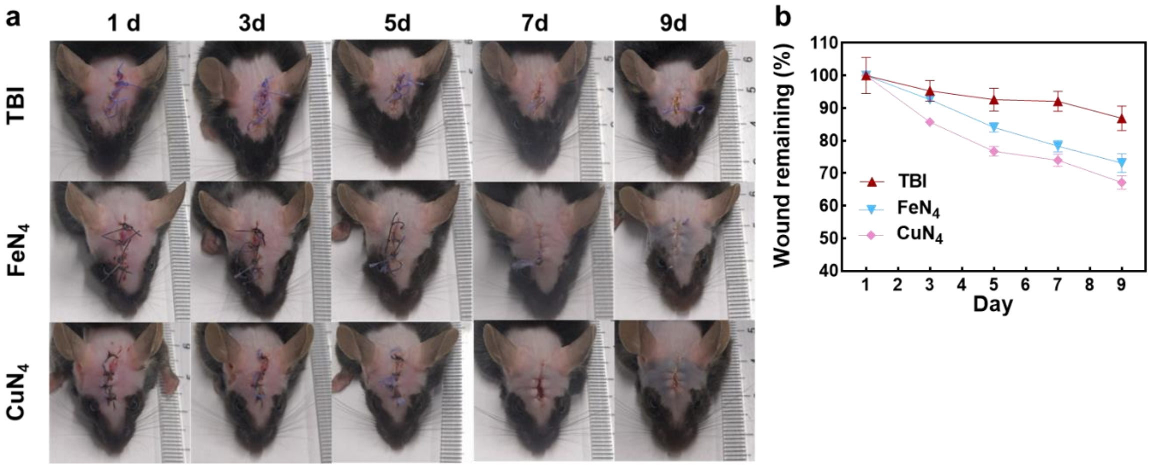

Supplementary Fig. 40. Representative photographs of scalp healing for living mice with traumatic brain injuries (TBI). (**a**) Residual wounds at different time points after treatment. (**b**) The remaining wound percentages over time after treatment of FeN_4 and CuN_4 sutures (n=3 images/3 mice, data are presented as mean ± standard error of the mean). Some error bars are too small to be visible.

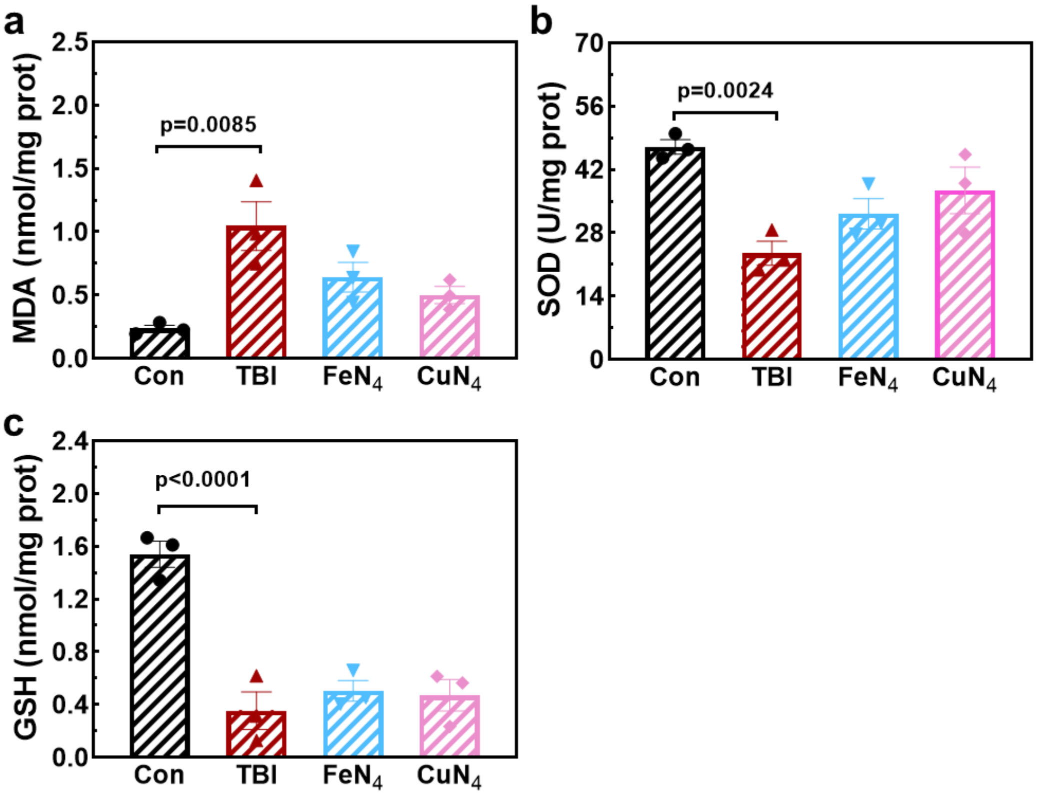

Supplementary Fig. 41. Indicators for oxidative stress. (**a**) MDA, (**b**) SOD, and (**c**) GSH of the scalp with or without MN_x sutures (M=Fe or Cu) on day 3 post-injury (n=3 biologically independent samples). Data were presented as mean ± standard error of the mean for triplicate measurements and compared with the TBI model group by one-way ANOVA with the one-sided Tukey's multiple comparisons test (the *p* values are shown). Differences with *p* values < 0.05 are considered significant.

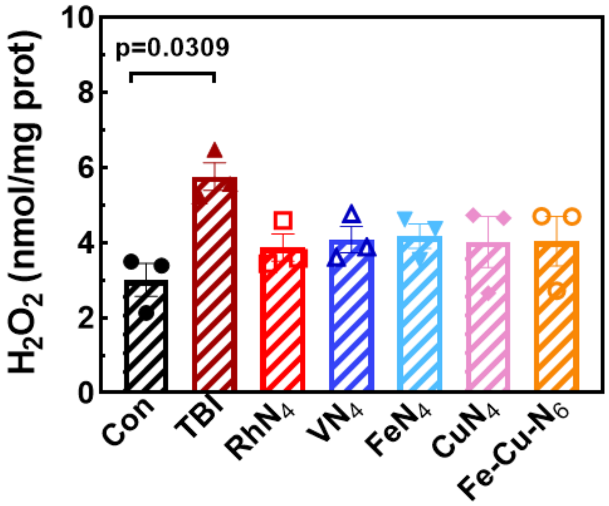

Supplementary Fig. 42. H_2O_2 levels in the scalp with or without MN_x sutures on day 3 post-injury (n=3 biologically independent samples). Data were presented as mean ± standard error of the mean for triplicate measurements and compared with the TBI model group by one-way ANOVA with the one-sided Tukey's multiple comparisons test (the *p* values are shown). Differences with *p* values < 0.05 are considered significant.

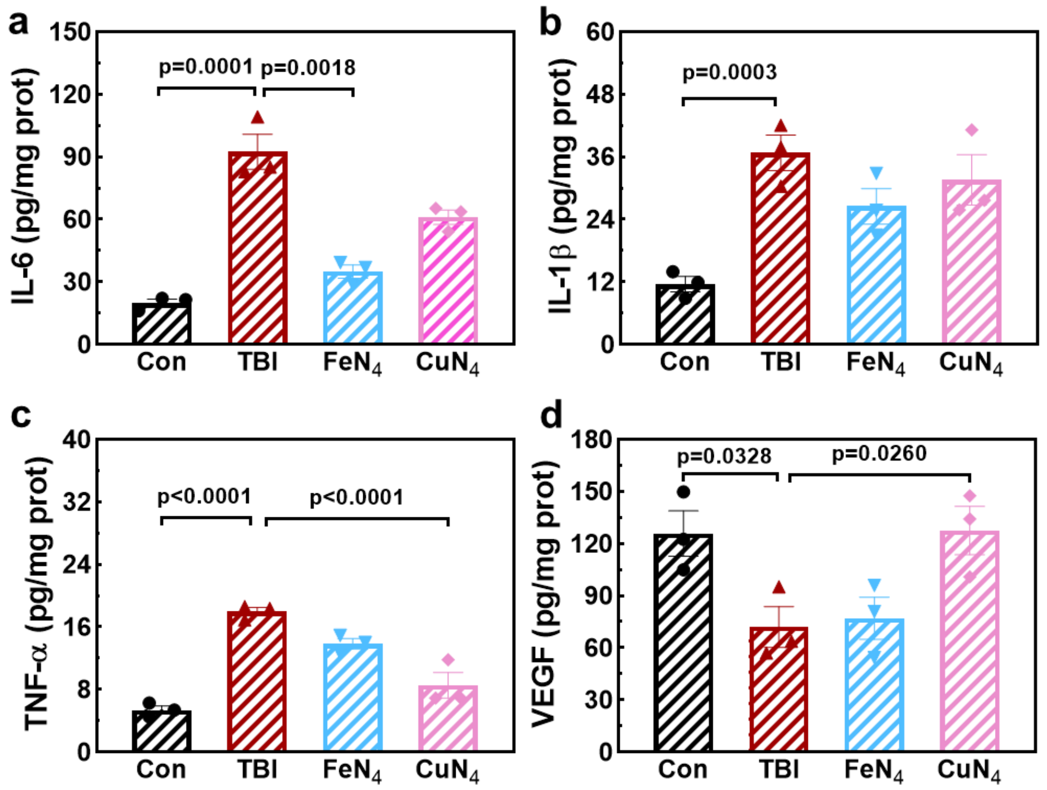

Supplementary Fig. 43. Quantitative ELISA assays of (**a**) IL-6, (**b**) IL-1β, (**c**) TNF-α, and (**d**) VEGF levels in scalp tissues with or without MN_x sutures on day 3 post-injury (n= 3 biologically independent samples), respectively. Data were presented as mean ± standard error of the mean and compared with the TBI group by one-way ANOVA with the one-sided Tukey's multiple comparisons test (the *p* values are shown). Differences with *p* values < 0.05 are considered significant.

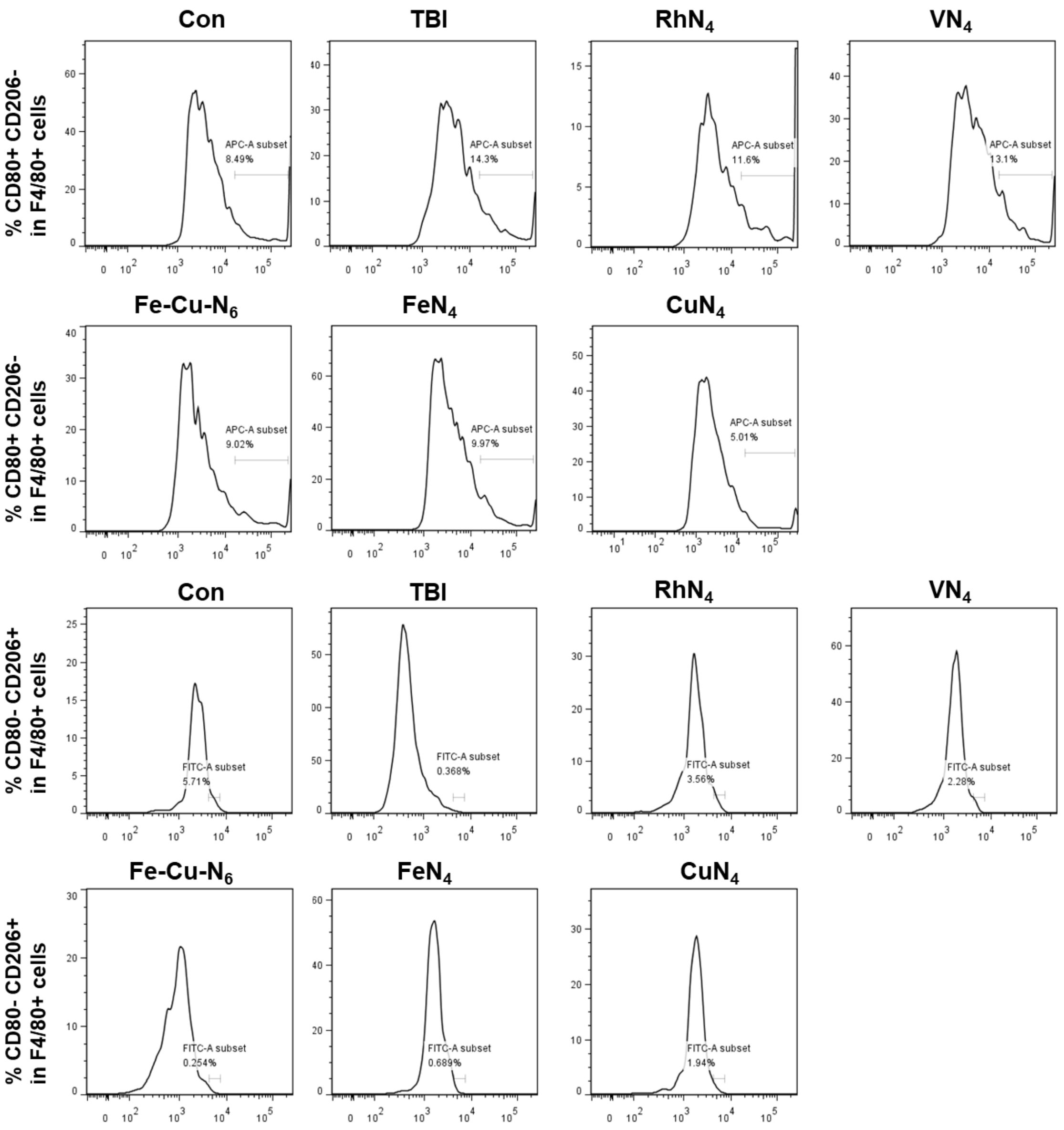

Supplementary Fig. 44. Representative gating strategy for analysis of M1 and M2 macrophages about flow cytometry.

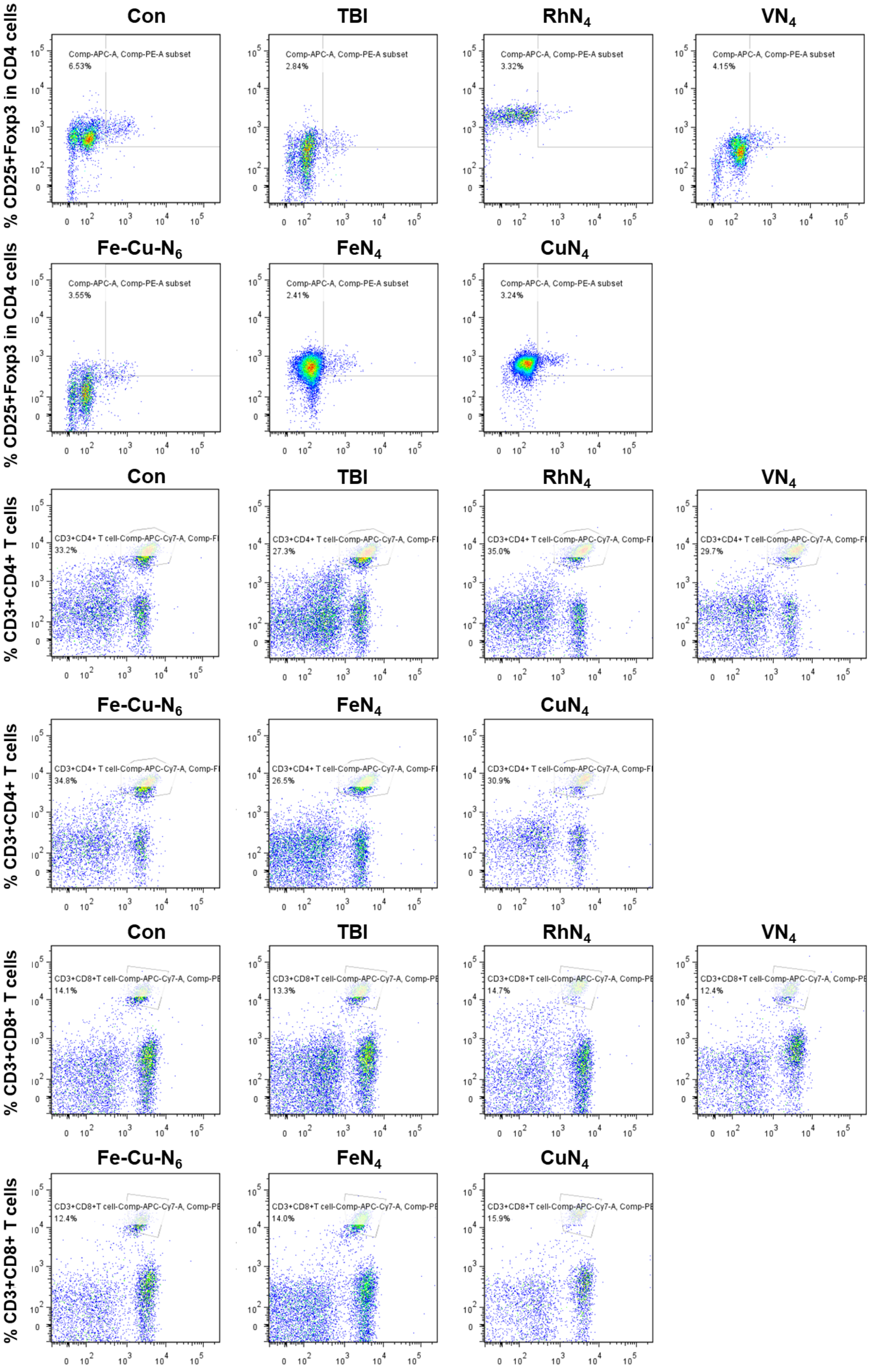

Supplementary Fig. 45. Representative gating strategy for analysis of Tregs, CD3+CD4+ T cells and CD3+CD8+ T cells about flow cytometry.

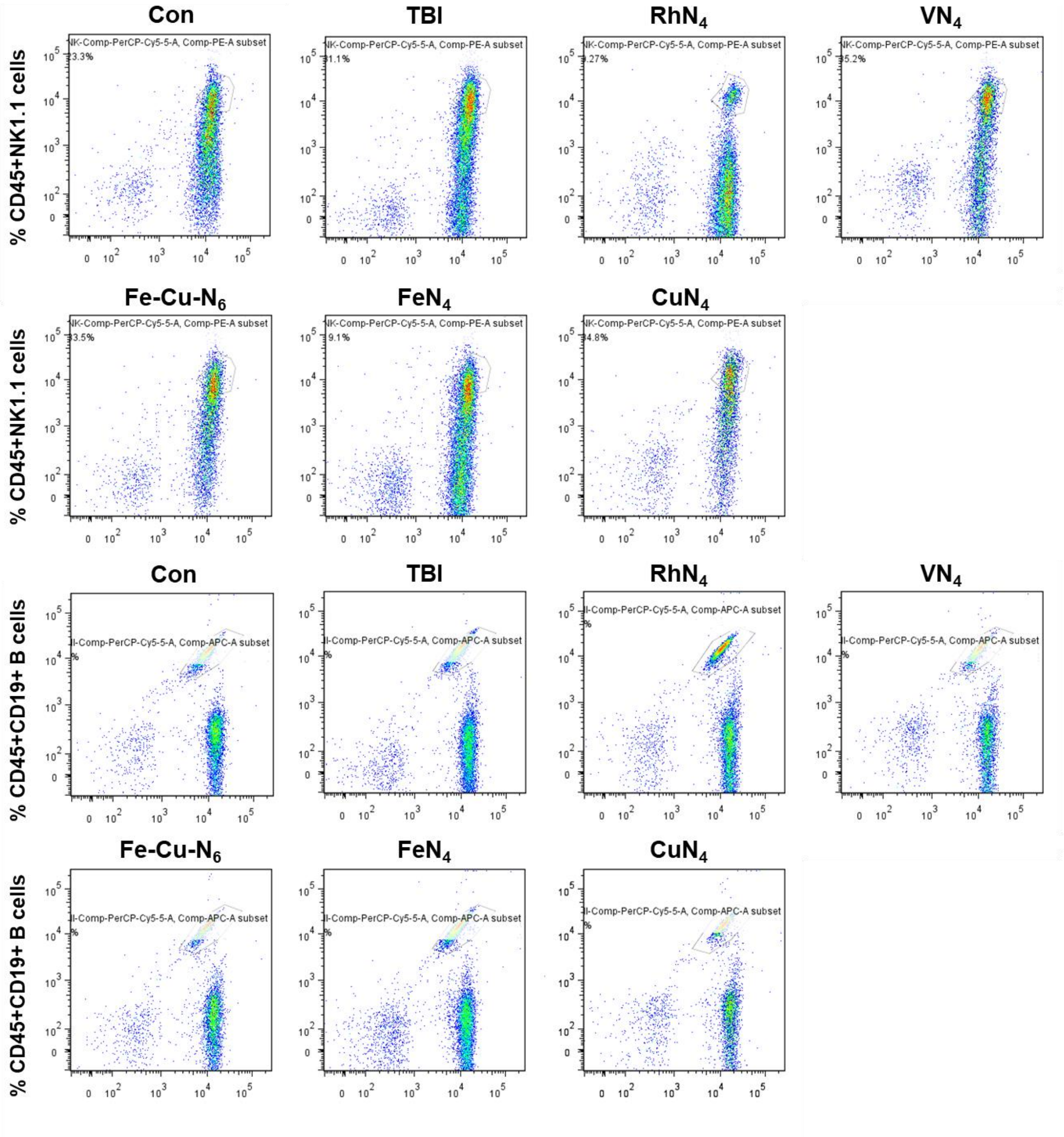

Supplementary Fig. 46. Representative gating strategy for analysis of NK and B cells about flow cytometry.

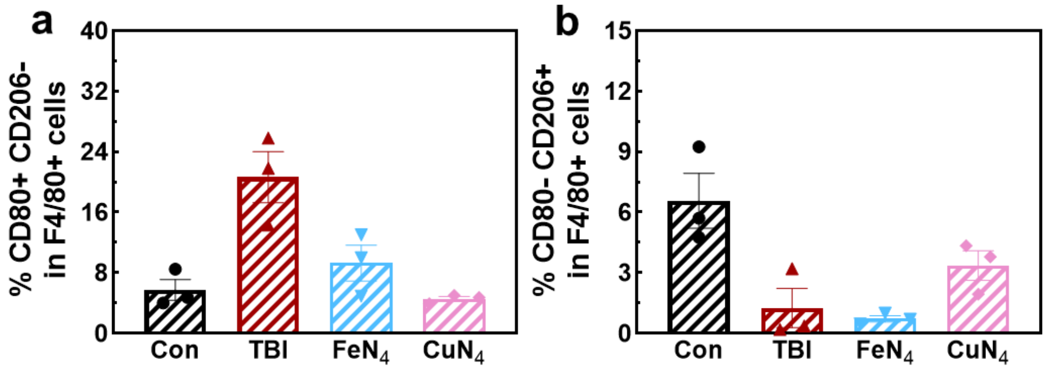

Supplementary Fig. 47. The flow cytometry analysis of (**a**) M1 and (**b**) M2 macrophages in the scalp after FeN_4 and CuN_4 treatments (n=3 biologically independent samples). Data were presented as mean ± standard error of the mean and compared with the TBI group by one-way ANOVA with the one-sided Tukey's multiple comparisons test. No statistically significant difference.

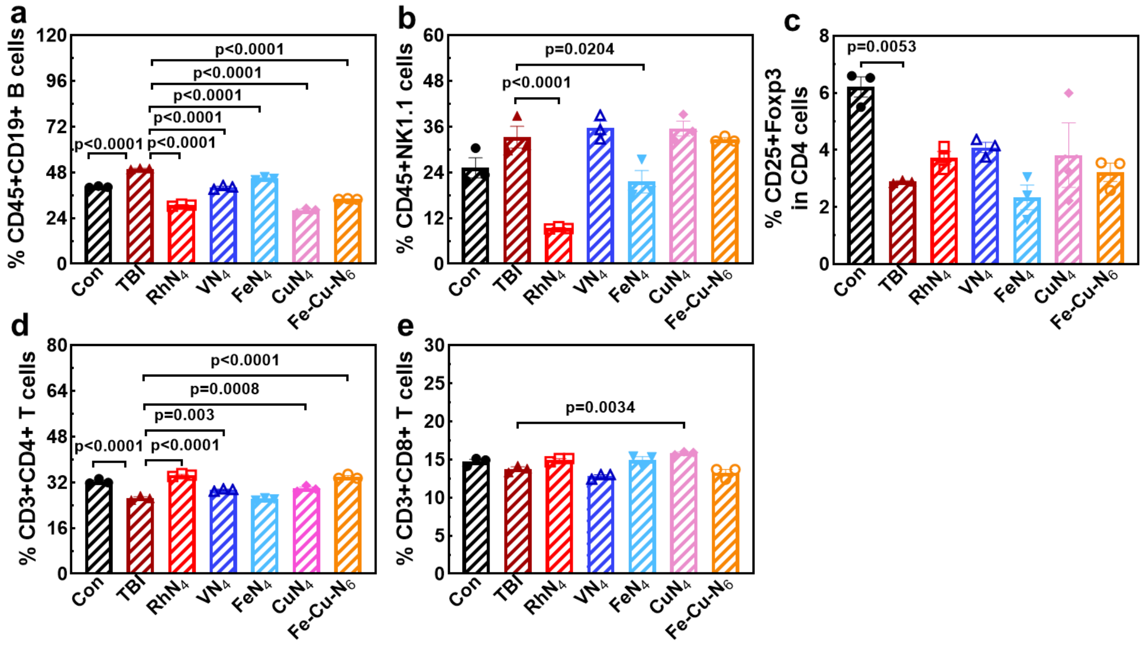

Supplementary Fig. 48. The flow cytometry analysis of (**a**) B cells, (**b**) NK cells, (**c**) Tregs, and (**d**) CD3+CD4+ T cells and (**e**) CD3+CD8+ T cells in the blood after various treatments (n=3 biologically independent samples). Data were presented as mean ± standard error of the mean and compared with the TBI group by one-way ANOVA with the one-sided Tukey's multiple comparisons test (the *p* values are shown). Differences with *p* values < 0.05 are considered significant.

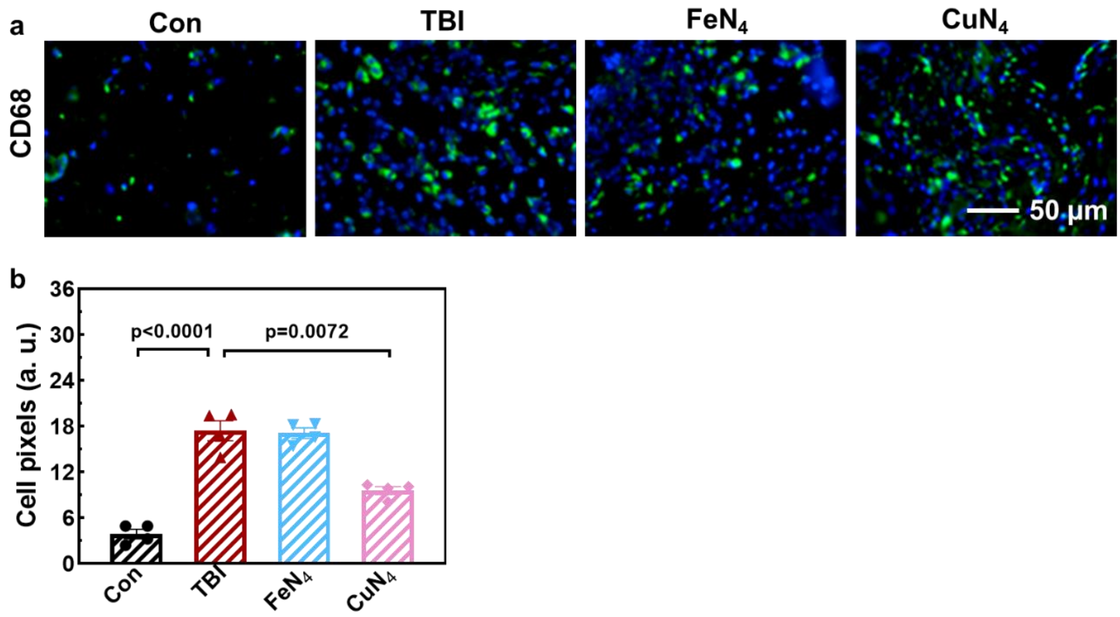

Supplementary Fig. 49. (**a**) Immunofluorescence (IF) staining of CD68 and (**b**) corresponding quantitative analysis for the injured scalps on day 4 post-TBI in the FeN_4 and CuN_4 groups (n= 4 images/ 4 mice). Data were presented as mean ± standard error of the mean and compared with the TBI group by one-way ANOVA with the one-sided Tukey's multiple comparisons test (the *p* values are shown). Differences with *p* values < 0.05 are considered significant. 'a. u.' represents arbitrary units.

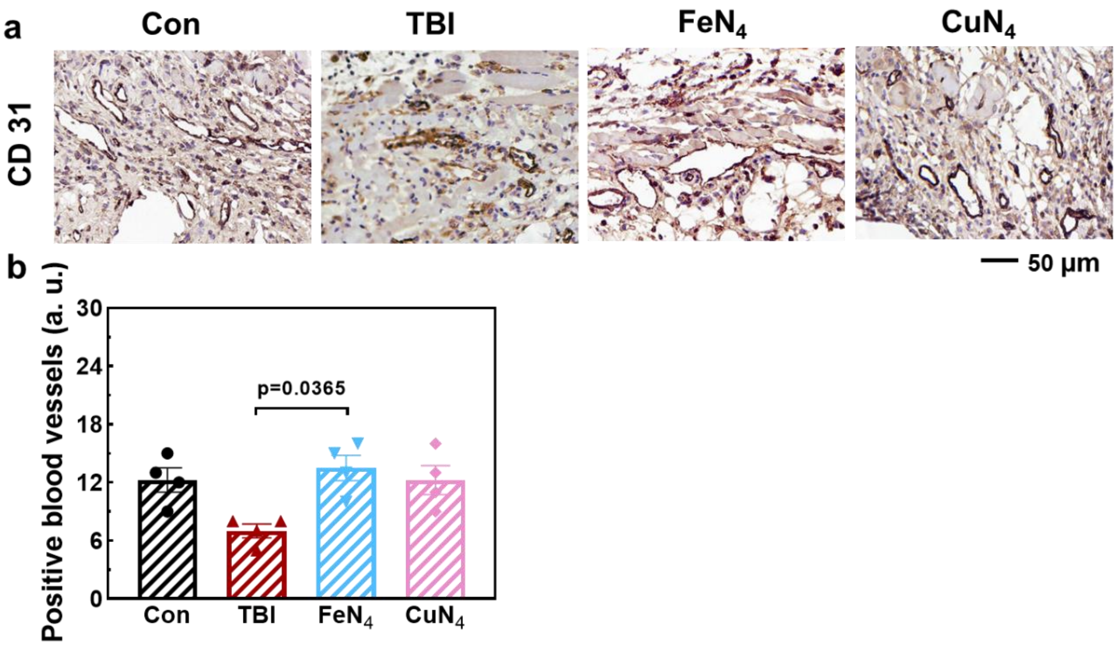

Supplementary Fig. 50. (**a**) Immunohistochemistry (IHC) staining of CD31 and (**b**) the corresponding quantitative analysis for scalp tissues on day 4 post-injury (n= 4 images/ 4mice). Data were presented as mean ± standard error of the mean and compared with the TBI group by one-way ANOVA with the one-sided Tukey's multiple comparisons test (the *p* values are shown). Differences with *p* values < 0.05 are considered significant. 'a. u.' represents arbitrary units.

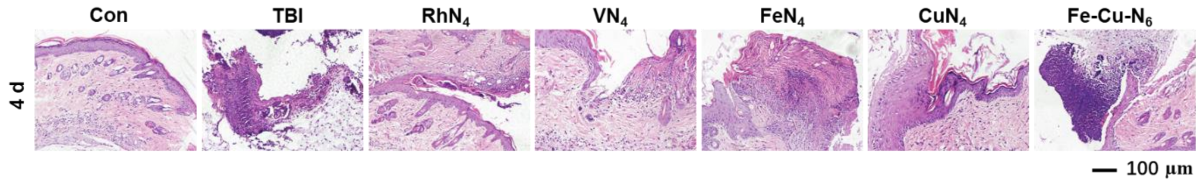

Supplementary Fig. 51. Hematoxylin and eosin (H&E) staining of scalp wound tissues on day 4 post-injury with or without sutures (n=4 images/4 mice).

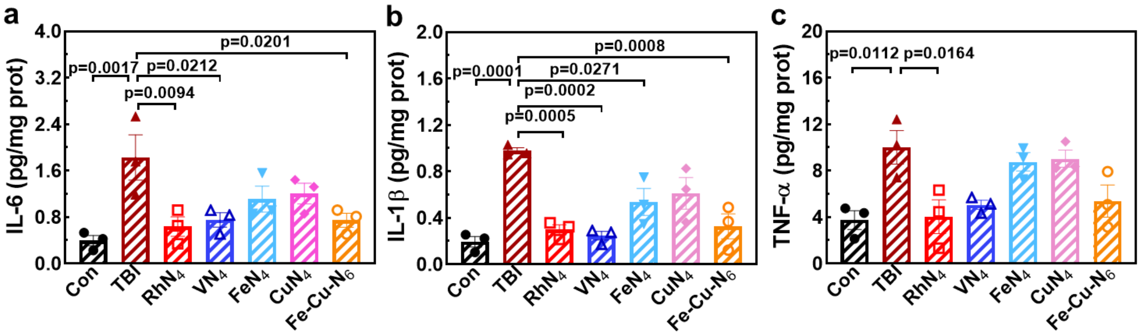

Supplementary Fig. 52 . Quantitative ELISA assays of (**a**) IL-1β, (**b**) IL-6, and (**c**) TNF-α in brain tissues on day 3 following brain injuries with or without MN_x sutures (n= 3 biologically independent samples). Data were presented as mean ± standard error of the mean and compared with the TBI groups by one-way ANOVA with the one-sided Tukey's multiple comparisons test (the *p* values are shown). Differences with *p* values < 0.05 are considered significant.

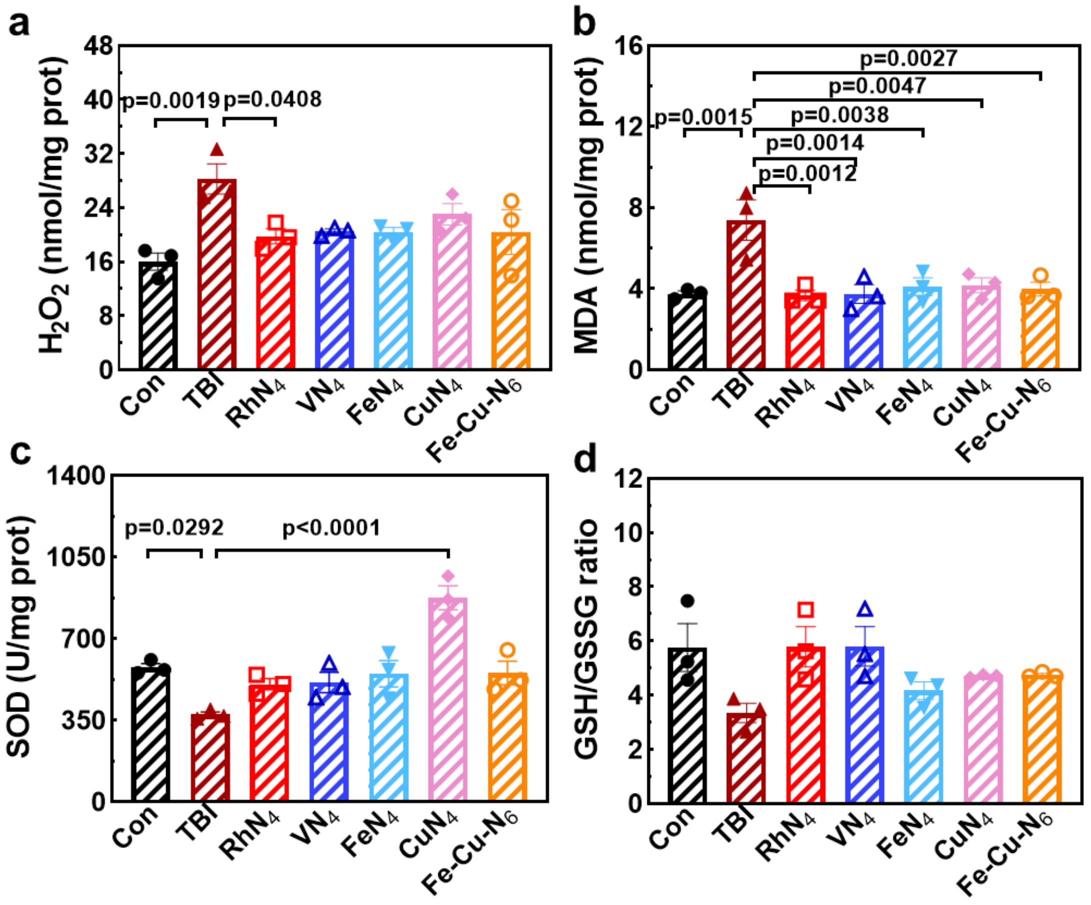

Supplementary Fig. 53. Indicators for oxidative stress, including (**a**) H_2O_2, (**b**) MDA, (**c**) SOD, and (**d**) the GSH/GSSG ratio of brain tissues with or without MN_x sutures on day 3 post-injury (n=3 biologically independent samples). Data were presented as mean ± standard error of the mean and compared with the TBI group by one-way ANOVA with the one-sided Tukey's multiple comparisons test (the *p* values are shown). Differences with *p* values < 0.05 are considered significant.

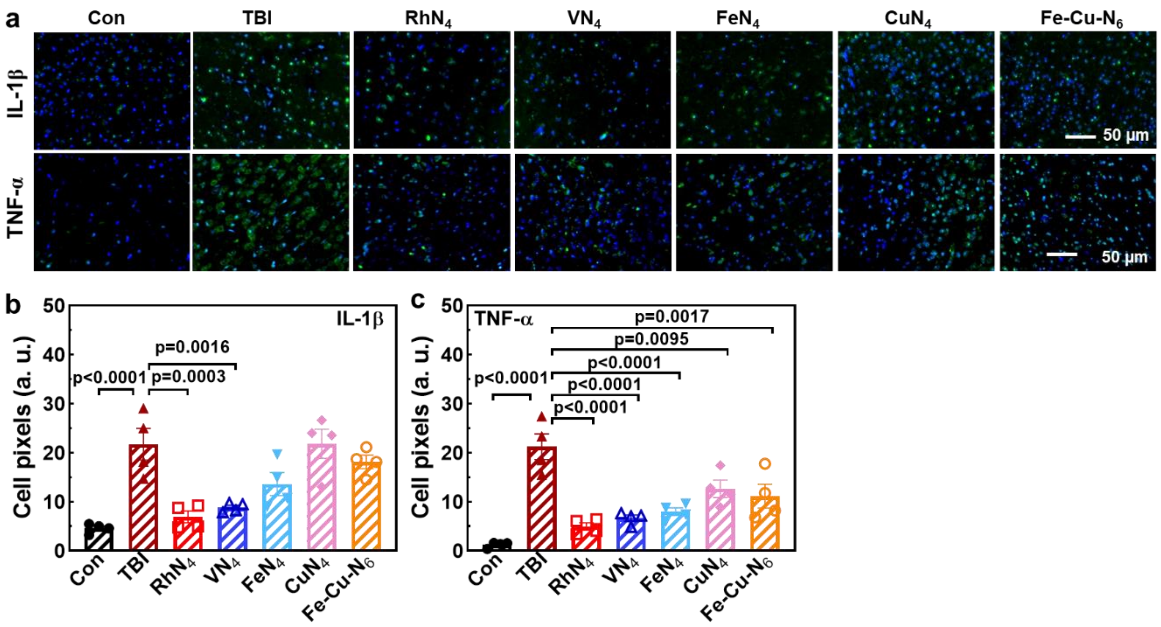

Supplementary Fig. 54. (**a**) IF staining and the corresponding quantitative analysis of (**b**) IL-1β and (**c**)TNF-α in the injured cortex on day 4 post brain injuries with or without MN_x sutures (n=4 images/ 4 mice). Data were presented as mean ± standard error of the mean and compared with the TBI group by one-way ANOVA with the one-sided Tukey's multiple comparisons test (the *p* values are shown). Differences with *p* values < 0.05 are considered significant. 'a. u.' represents arbitrary units.

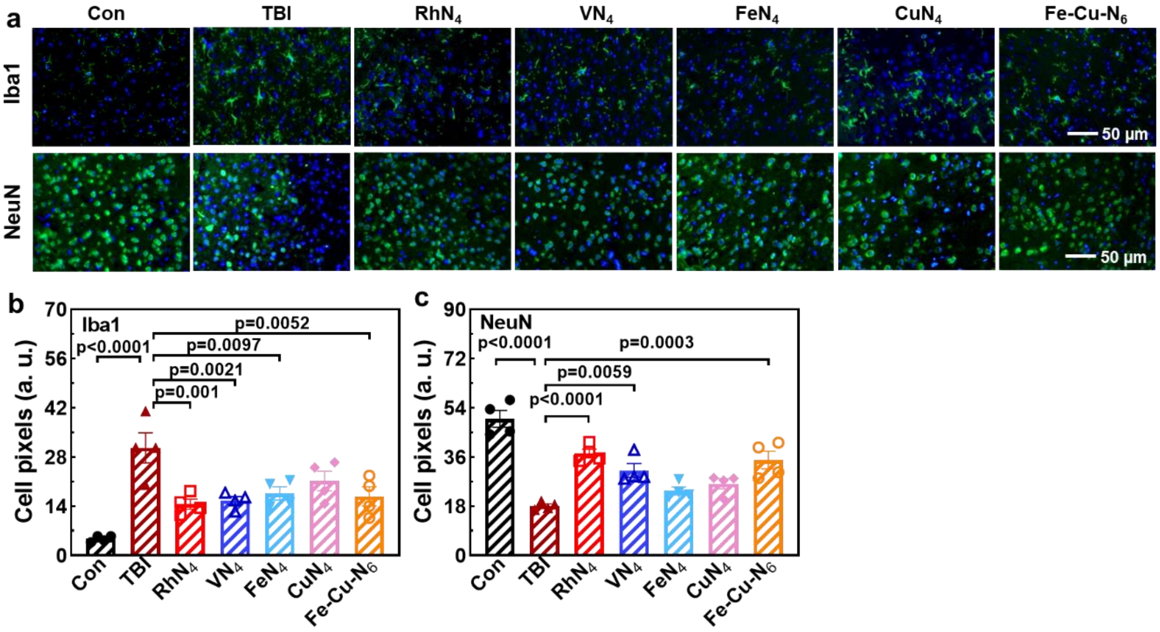

Supplementary Fig. 55. (**a**) IF staining of ionized calcium-binding adaptor molecule 1 (Iba1) for microglia and the neuronal nuclear protein for neurons (NeuN) in the injured cortex of mice on day 4 post brain injuries with or without MN_x sutures (n=4 per group). (**b, c**) Associated quantitative analysis. Data were presented as mean ± standard error of the mean and compared with the TBI group by one-way ANOVA with the one-sided Tukey's multiple comparisons test (the *p* values are shown). Differences with *p* values < 0.05 are considered significant. 'a. u.' represents arbitrary units.

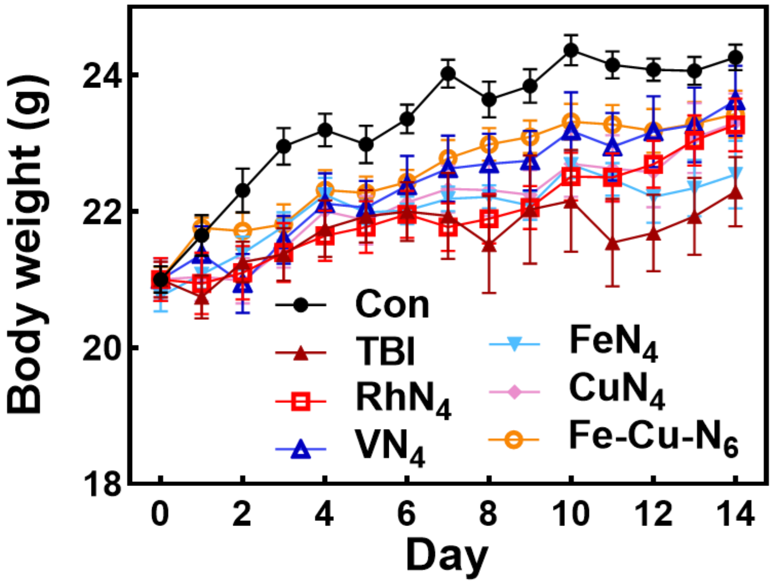

Supplementary Fig. 56. Body weight changes over time in all groups with or without treatment of MN_x sutures. Data were presented as mean ± standard error of the mean (n=7 mice).

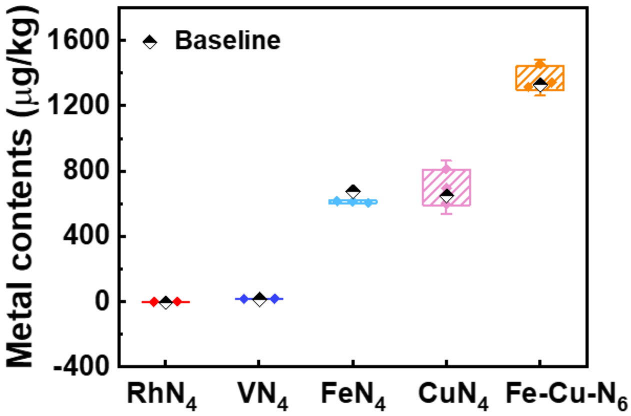

Supplementary Fig. 57. Metal content in the blood in the MN_x-treated group on day 6 post-injury (n=3 mice, boxes represent the median and IQR and the upper and lower whiskers extending to the values that are within 1.5 × IQR). The baseline represents the corresponding metal content in the blood of normal mice.

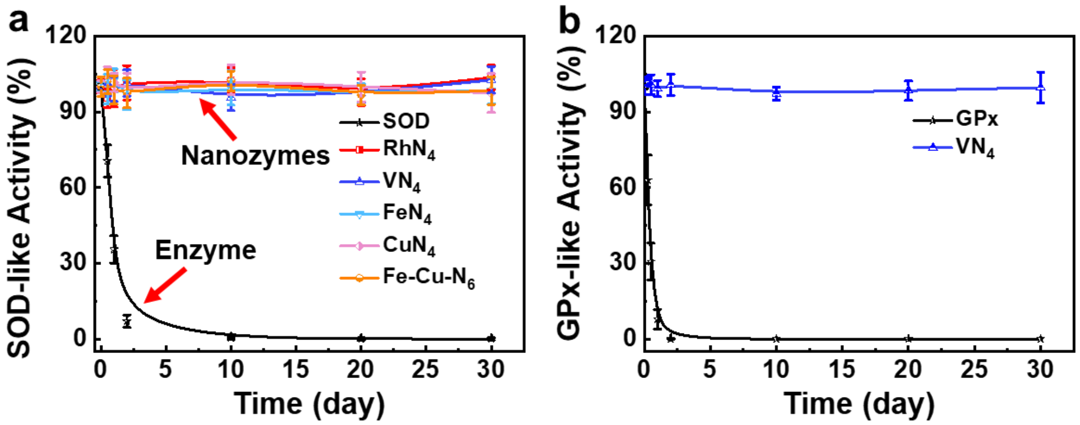

Supplementary Fig. 58. Comparison of the stability of (**a**) SOD- and (**b**) GPx-like activities for MN_x and the natural enzymes (n=3 independent experiments, data are presented as mean ± SD). All tests were performed at room temperature.

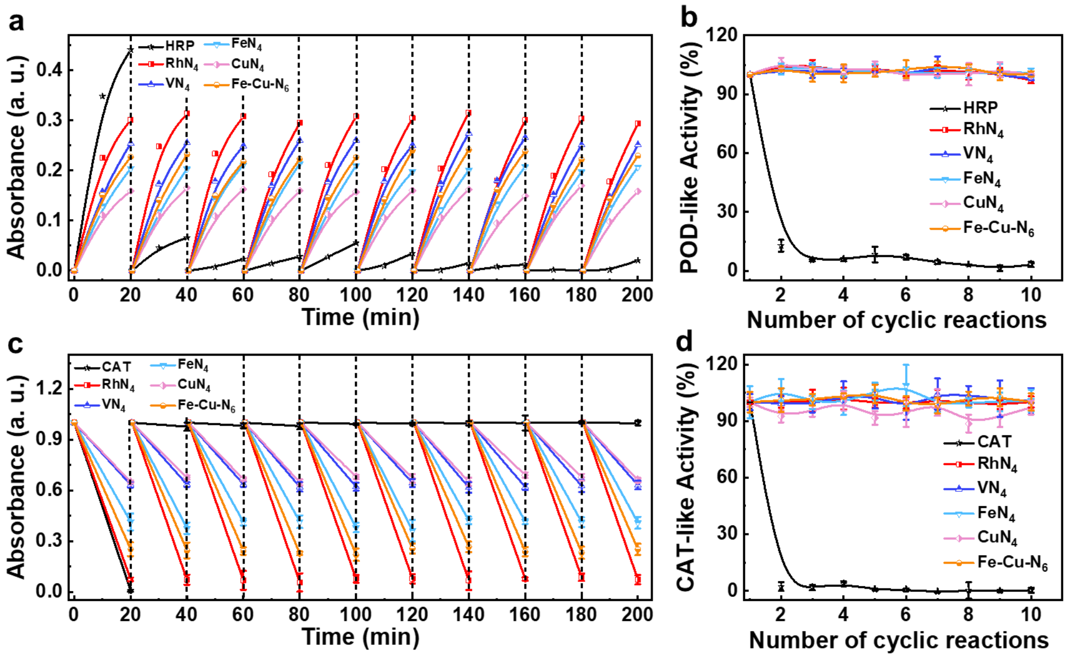

Supplementary Fig. 59. Comparison of the recyclability of (**a, b**) POD-, (**c, d**) CAT-like activities for MN_x and the natural enzymes (n=3 independent experiments, data are presented as mean ± SD). Natural enzymes almost lost all the enzyme activities after one catalysis cycle, while MN_x could be recycled multiple times without apparently losing activities. All tests were performed at room temperature. ‘a. u.’ represents arbitrary units.

Supplementary Table 1. Quantification for metal contents of active sites by inductively coupled plasma-mass spectrometry (ICP-MS).

Catalysts	Rh (wt.%)	V (wt.%)	Fe (wt.%)	Cu (wt.%)
RhN_4	0.19			
VN_4		0.22		
FeN_4			1.4	
CuN_4				1.39
Fe-Cu-N_6			0.54	0.56

Supplementary Table 2. Structural parameters of FeN_4, CuN_4, and Fe-Cu-N_6 obtained from Fe K-edge and Cu K-edge EXAFS by curve fitting.

Catalysts	Path	*N*	*R* (Å)	σ^2 (10^{-3}Å^2)	ΔE_0 (eV)	*R factor*
FeN_4	Fe-N	4.0	1.97	7.2±0.3	6.8±1.2	0.02
CuN_4	Cu-N	4.0	1.96	6.2±0.7	-1.8±0.6	0.002
Fe-Cu-N_6	Fe-N	4.0	1.97	7.1±0.2	9.2±1.1	0.014
Fe-Cu-N_6	Cu-N	4.0	1.96	5.2±0.6	-0.9±1.0	0.002

N, coordination number; *R*, bonding distance between central atoms and surrounding coordination atoms; σ^2, Debye-Waller factor; ΔE_0, inner potential shift; *R factor* represents the goodness of the fitting.

Supplementary Table 3. The formation energies and the distance of Fe-Cu in five different Fe-Cu-N_x structures.

Structures	Fe-Cu-N_6	Fe-Cu-N_8-I	Fe-Cu-N_8-II	Fe-Cu-N_8-III	Fe-Cu-N_8-IV
Formation energy (eV)	-5.432	-3.559	-4.285	-4.164	-2.967
Distance of Fe-Cu (Å)	2.46	5.04	4.36	4.86	4.04

Supplementary Table 4. Comparison of the kinetic parameters between MN_x and HRP towards the TMB substrate during the POD-mimic catalysis.

Catalysts	[E] (M)	Substance	K_m (μM)	V_m (μM min^{-1})	k_{cat} (min^{-1})
RhN_4	5.61×10^{-8}	TMB	55.7	5.88	1.05×10^{2}
VN_4	1.28×10^{-7}	TMB	65.6	7.00	0.547×10^{2}
FeN_4	7.50×10^{-7}	TMB	184.6	12.34	0.165×10^{2}
CuN_4	6.57×10^{-7}	TMB	245.7	4.83	0.073×10^{2}
$Fe\text{-}Cu\text{-}N_6$	5.55×10^{-7}	TMB	177.4	22.9	0.416×10^{2}
HRP	2.00×10^{-10}	TMB	276.2	4.03	2.01×10^{4}

[E], the molar concentration of the active sites; K_m, the Michaelis constant; V_m, maximal reaction velocity; k_{cat}, catalytic constant, where $k_{cat} = V_m/[E]$.

Supplementary Table 5. Comparison of the kinetic parameters between MN_x and HRP towards the H_2O_2 substrate during the POD-mimic catalysis.

Catalysts	[E] (M)	Substance	K_m (μM)	V_m (μM min^{-1})	k_{cat} (min^{-1})
RhN_4	5.61×10^{-8}	H_2O_2	12.38	5.08	0.903×10^{2}
VN_4	1.28×10^{-7}	H_2O_2	14.18	7.92	0.619×10^{2}
FeN_4	7.50×10^{-7}	H_2O_2	12.45	15.05	0.201×10^{2}
CuN_4	6.57×10^{-7}	H_2O_2	18.55	3.84	0.058×10^{2}
Fe-Cu-N_6	5.55×10^{-7}	H_2O_2	14.47	21.15	0.381×10^{2}
HRP	2.00×10^{-10}	H_2O_2	2.63	1.97	9.85×10^{3}

[E], the molar concentration of the active sites; K_m, the Michaelis constant; V_m, maximal reaction velocity; k_{cat}, catalytic constant, where $k_{cat} = V_m$/[E].

Supplementary Table 6. The energy barriers of POD processes for five different Fe-Cu-N_x bimetallic structures.

Structures	Fe-Cu-N_6	Fe-Cu-N_8-I	Fe-Cu-N_8-II	Fe-Cu-N_8-III	Fe-Cu-N_8-IV
Energy barrier (eV)	0.479	1.020	1.121	1.691	1.824

Supplementary Table 7. The energy barriers of POD processes with and without generating •OH of MN_x.

Catalysts		RhN_4	VN_4	FeN_4	CuN_4	Fe-Cu-N_6
Energy barrier (eV)	•OH-producing	1.120	3.427	0.405	2.447	1.244
	non-•OH-producing	0.213	0.226	0.539	1.044	0.479

Supplementary Table 8. Comparison of the kinetic parameters between MN_x and CAT towards H_2O_2 substrate during CAT-mimic catalysis.

Catalysts	[E] (M)	Substance	K_m (mM)	V_m (μM min^{-1})	k_{cat} (min^{-1})
RhN_4	5.61×10^{-8}	H_2O_2	1.33	30.0	5.35×10^{2}
VN_4	1.28×10^{-7}	H_2O_2	2.92	9.11	0.712×10^{2}
FeN_4	7.50×10^{-7}	H_2O_2	2.04	55.4	0.739×10^{2}
CuN_4	6.57×10^{-7}	H_2O_2	3.17	19.9	0.303×10^{2}
Fe-Cu-N_6	5.55×10^{-7}	H_2O_2	1.99	63.0	1.13×10^{2}
CAT	6.00×10^{-9}	H_2O_2	28.8	1.04×10^{4}	1.73×10^{4}

[E], the molar concentration of the metal activation sites; K_m, Michaelis constant; V_m, maximal reaction velocity; k_{cat}, catalytic constant, where $k_{cat} = V_m/[E]$.

Supplementary Table 9. Comparison of the kinetic parameters between VN_4 and GPx towards GSH and H_2O_2 substrate during enzymatic catalysis.

Catalysts	[E] (M)	Substance	K_m (mM)	V_m (μM min^{-1})	k_{cat} (min^{-1})
VN_4	8.58×10^{-7}	GSH	1.31	8.55	10.0
		H_2O_2	0.050	7.90	9.21
GPx	1.82×10^{-9}	GSH	9.23	46.8	2.57×10^{4}
		H_2O_2	0.042	14.1	7.75×10^{3}

[E], the molar concentration of the metal activation sites; K_m, Michaelis constant; V_m, maximal reaction velocity; k_{cat}, catalytic constant, where $k_{cat} = V_m/[E]$.

Supplementary Table 10. Elemental quantification of the metal active sites in corresponding MN_x sutures determined by ICP-MS.

Catalysts	Rh (wt.%)	V (wt.%)	Fe (wt.%)	Cu (wt.%)
RhN_4	0.011			
VN_4		0.018		
FeN_4			0.013	
CuN_4				0.012
$Fe\text{-}Cu\text{-}N_6$			0.0065	0.0058
