# Supplementary material for: Single-atom nanozymes catalytically surpassing naturally occurring enzymes as sustained stitching for brain trauma
Source: Nat Commun. 2022 Aug 12;13:4744. doi: 10.1038/s41467-022-32411-z (PMC9374753; doi:10.1038/s41467-022-32411-z)
Supplement: Supplementary file 3 — Reporting Summary [file 41467_2022_32411_MOESM3_ESM.pdf]

nature portfolio

Corresponding author(s): Xiao-Dong Zhang

Last updated by author(s): Jul 3, 2022

Reporting Summary

Nature Portfolio wishes to improve the reproducibility of the work that we publish. This form provides structure for consistency and transparency in reporting. For further information on Nature Portfolio policies, see our Editorial Policies and the Editorial Policy Checklist.

Statistics

For all statistical analyses, confirm that the following items are present in the figure legend, table legend, main text, or Methods section.

n/a	Confirmed	
☐	☒	The exact sample size (n) for each experimental group/condition, given as a discrete number and unit of measurement
☐	☒	A statement on whether measurements were taken from distinct samples or whether the same sample was measured repeatedly
☐	☒	The statistical test(s) used AND whether they are one- or two-sided<br>*Only common tests should be described solely by name; describe more complex techniques in the Methods section.*
☒	☐	A description of all covariates tested
☐	☒	A description of any assumptions or corrections, such as tests of normality and adjustment for multiple comparisons
☐	☒	A full description of the statistical parameters including central tendency (e.g. means) or other basic estimates (e.g. regression coefficient) AND variation (e.g. standard deviation) or associated estimates of uncertainty (e.g. confidence intervals)
☐	☒	For null hypothesis testing, the test statistic (e.g. F, t, r) with confidence intervals, effect sizes, degrees of freedom and P value noted<br>*Give P values as exact values whenever suitable.*
☒	☐	For Bayesian analysis, information on the choice of priors and Markov chain Monte Carlo settings
☒	☐	For hierarchical and complex designs, identification of the appropriate level for tests and full reporting of outcomes
☒	☐	Estimates of effect sizes (e.g. Cohen's d, Pearson's r), indicating how they were calculated

Our web collection on statistics for biologists contains articles on many of the points above.

Software and code

Policy information about availability of computer code

Data collection	The data were collected on the field-emission transmission electron microscopes (JEM-F200 and JEM-2100F, JEOL, Japan), emission spherical aberration-corrected atomic resolution microscope (JEM-ARM200F, JEOL, Japan), field-emission scanning electron microscope (S-4800, Hitachi, Japan), 1W1B station (Beijing Synchrotron Radiation Facility), UV-VIS NIR instrument (Shimadzu, Japan), X-ray powder diffractometer (Smartlab, Rigaku, Japan), ultra-fast Raman imaging spectrometer (XploRA PLUS, Horiba-JY, France), X-ray photoelectron spectrometer (ESCALAB Xi+, Thermo Fisher Scientific, UK), ICP-MS measurement (7900 ICP-MS, Agilent, UK), Electron spin resonance spectrometer (JES-FA200, JEOL, Japan), and Flow cytometry (BD FACSCanto II).
Data analysis	In this study, Microsoft Excel 2016, Origin 9.0/2021, Gaussian 09 package, ARTEMIS, IFEFFIT, XPSPEAK41, ImageJ 1.4.3.67 , Graphpad 8, DigitalMicrograph, and Flowjo 10.6.2 were used to analyzed the data.

For manuscripts utilizing custom algorithms or software that are central to the research but not yet described in published literature, software must be made available to editors and reviewers. We strongly encourage code deposition in a community repository (e.g. GitHub). See the Nature Portfolio guidelines for submitting code & software for further information.

Data

Policy information about availability of data

All manuscripts must include a data availability statement. This statement should provide the following information, where applicable:

- Accession codes, unique identifiers, or web links for publicly available datasets
- A description of any restrictions on data availability
- For clinical datasets or third party data, please ensure that the statement adheres to our policy

All data supporting the findings of this study are available within this article and Supplementary Information files. Source data are provided with this paper. Data is available from the corresponding authors upon request.

Field-specific reporting

Please select the one below that is the best fit for your research. If you are not sure, read the appropriate sections before making your selection.

- [x] Life sciences
- [ ] Behavioural & social sciences
- [ ] Ecological, evolutionary & environmental sciences

For a reference copy of the document with all sections, see nature.com/documents/nr-reporting-summary-flat.pdf

Life sciences study design

All studies must disclose on these points even when the disclosure is negative.

Sample size	No sample size calculation was performed. All biologically based assays were performed with the usual and sufficient sample size setting determined by previous experiments, for example, Nat Commun 12, 114 (2021). These sample sizes were sufficient for a statistical analysis. All experiments reported have n number and repetitions reported.
Data exclusions	No data was excluded from the analysis.
Replication	Results shown in the manuscript are representative of at least two similar experiments. All our attempts at replication were successful.
Randomization	Our samples/ organisms were allocated randomly.
Blinding	In all experiments, investigators were blinded to group allocation during data collection and processing.

Reporting for specific materials, systems and methods

We require information from authors about some types of materials, experimental systems and methods used in many studies. Here, indicate whether each material, system or method listed is relevant to your study. If you are not sure if a list item applies to your research, read the appropriate section before selecting a response.

Materials & experimental systems

n/a	Involved in the study	
☐	☒	Antibodies
☒	☐	Eukaryotic cell lines
☒	☐	Palaeontology and archaeology
☐	☒	Animals and other organisms
☒	☐	Human research participants
☒	☐	Clinical data
☒	☐	Dual use research of concern

Methods

n/a	Involved in the study	
☒	☐	ChIP-seq
☐	☒	Flow cytometry
☒	☐	MRI-based neuroimaging

Antibodies

Antibodies used	Antibodies used:<br>CD31 Polyclonal antibody, Proteintech, 28083-1-AP. Lot: 00098789, source: rabbit.<br>CD68 Polyclonal antibody, Proteintech, 28058-1-AP. Lot: 00097019, source: rabbit.<br>Anti-TNFα antibody, Abcam, ab183218. Lot: GR284782-19, source: rabbit.<br>Anti-IL-1β antibody, Affinity, AF5103. Lot: 63h9328, source: rabbit.<br>Anti-Iba1 antibody, Abcam, ab48004. Lot: GR123692-54, source: rabbit.<br>Anti-NeuN antibody, Proteintech, 26975-1-AP. Lot: 00050791, source: rabbit.<br>Alexa Fluor 488 – conjugated Affinipure Goat Anti-Rabbit IgG(H+L), Proteintech, SA00006-2. Lot: 20000098<br>PE anti-mouse F4/80 Antibody, Biolegend, 123110, Lot: B340064, stock solution.

	APC anti-mouse CD80, Biolegend, 104714, Lot: B342621, stock solution.<br>FITC anti-mouse CD206 (MMR) Antibody, Biolegend, 141704, Lot: B350306, stock solution.<br>APC/Cyanine7 anti-mouse CD3 Antibody, Biolegend, 100222, Lot: B324939, stock solution.<br>PE/Cyanine7 anti-mouse CD8a, Biolegend, 100722, Lot: B329645, stock solution.<br>FITC anti-mouse CD4 Antibody, Biolegend, 100406, Lot: B351029, stock solution.<br>PE anti-mouse NK-1.1 Antibody, Biolegend, 156504, Lot: B306542, stock solution.<br>PerCp anti-mouse CD45, Biolegend, 103130, Lot: B330011, stock solution.<br>APC anti-mouse CD19, Biolegend, 152410, Lot: B321732, stock solution.<br>APC anti-mouse CD25 Antibody, Biolegend, 101909, Lot: B317107, stock solution.<br>PE-Foxp3 Monoclonal Antibody (FJK-16s), Thermo, 12-5773-82, stock solution.<br>Rat IgG2a kappa Isotype Control, Invitrogen, 12-4321-82, Lot: 2389552, stock solution.<br>PE Rat IgG2a, κ Isotype Ctrl Antibody, Biolegend, 400508, Lot: B34004, stock solution.<br>Anti-Mo/Rt Foxp3, Invitrogen, 12-5773-82, Lot:2344844, stock solution.
Validation	All the antibodies commercially purchased from these companies: link provided :1) https://www.abcam.cn/, 2) http://www.ptgcn.com/, 3) http://www.affbiotech.cn/, 4) https://www.biolegend.com/, 5) https://www.thermofisher.cn/cn/zh/home/brands/invitrogen.html, 6) https://www.thermofisher.cn/cn/zh/home.html?CID=ebz_bus_sbu_r04_cn_0se_360_pt_PUR_TFS_gene_aBrand_pc_mkt_050821_0000000AF3D7AB27. Each of the antibody can be searched by their clone numbers on their respective website. In general all of the antibodies were quality control tested by immunofluorescent staining with flow cytometric analysis by the companies.<br>CD31 Polyclonal antibody, Proteintech, 28083-1-AP, provided data in manuscript: IHC.<br>CD68 Polyclonal antibody, Proteintech, 28058-1-AP, provided data in manuscript: IF.<br>Anti-TNFα antibody, Abcam, ab183218, provided data in manuscript: IF.<br>Anti-IL-1β antibody, Affinity, AF5103, provided data in manuscript: IF.<br>Anti-Iba1 antibody, Abcam, ab48004, provided data in manuscript: IF.<br>Anti-NeuN antibody, Proteintech, 26975-1-AP, provided data in manuscript: IF.<br>Alexa Fluor 488 – conjugated Affinipure Goat Anti-Rabbit IgG(H+L), Proteintech, SA00006-2 provided data in manuscript: IF<br>PE anti-mouse F4/80 Antibody, Biolegend, 123110, provided data in manuscript: FC<br>APC anti-mouse CD80, Biolegend, 104714, provided data in manuscript: FC<br>FITC anti-mouse CD206 (MMR) Antibody, Biolegend, 141704, provided data in manuscript: FC<br>APC/Cyanine7 anti-mouse CD3 Antibody, Biolegend, 100222, provided data in manuscript: FC<br>PE/Cyanine7 anti-mouse CD8a, Biolegend, 100722, provided data in manuscript: FC<br>FITC anti-mouse CD4 Antibody, Biolegend, 100406, provided data in manuscript: FC<br>PE anti-mouse NK-1.1 Antibody, Biolegend, 156504, provided data in manuscript: FC<br>PerCp anti-mouse CD45, Biolegend, 103130, provided data in manuscript: FC<br>APC anti-mouse CD19, Biolegend, 152410, provided data in manuscript: FC<br>APC anti-mouse CD25 Antibody, Biolegend, 101909, provided data in manuscript: FC<br>PE-Foxp3 Monoclonal Antibody (FJK-16s), Thermo, 12-5773-82, provided data in manuscript: FC<br>Anti-Mo/Rt Foxp3, Invitrogen, 12-5773-82, Lot:2344844, provided data in manuscript: FC

Animals and other organisms

Policy information about studies involving animals; ARRIVE guidelines recommended for reporting animal research

Laboratory animals	C57BL/6J mice, male, 7-9 weeks. Mice were housed in a constant temperature (21–23 °C) and animal humidity (45–60%) environment with a 12-hour light-dark cycle. Food and water are available ad libitum.
Wild animals	The study did not involve wild animals.
Field-collected samples	No field-collected samples were used in this study.
Ethics oversight	The ethics oversight is approved by the Institute of Radiation Medicine, Chinese Academy of Medical Science, and Peking Union Medical College, following the ethics and ethics rules of the Animal Committee and complying with the principles of animal protection, animal welfare, and ethics (IRM-DWLI-2021107).

Note that full information on the approval of the study protocol must also be provided in the manuscript.

Flow Cytometry

Plots

Confirm that:

- [x] The axis labels state the marker and fluorochrome used (e.g. CD4-FITC).
- [x] The axis scales are clearly visible. Include numbers along axes only for bottom left plot of group (a 'group' is an analysis of identical markers).
- [x] All plots are contour plots with outliers or pseudocolor plots.
- [x] A numerical value for number of cells or percentage (with statistics) is provided.

Methodology

Sample preparation	To evaluate the changes of immune cells in the wound and blood, the scalp and blood were collected in different groups on day 6 post brain injury. The fresh wound tissues were digested by collagenase and hyaluronidase (Stemcell, 07919) overnight at 37 ^{o}C to obtain single cells for flow cytometry analysis (BD FACSCanto II). Lymphocytes in the blood were collected with Ficoll kits (Solarbio, P8620).
Instrument	A FACS flow cytometer (BD FACSCanto II)
Software	BD FACSCanto II
Cell population abundance	No cell sorting was performed in this work. 10^6 cells/mL. Cell counts were determined by hematocrit plates. Scalp samples were obtained by collagenase and hyaluronidase digestion. Blood cells were obtained by Ficoll kits (Solarbio, P8620).
Gating strategy	Cells were gated based on size and granularity by forward and side scatter (SSC versus FCS). Then, cell gate is analyzed for specific fluorescence.

- [x] Tick this box to confirm that a figure exemplifying the gating strategy is provided in the Supplementary Information.
